# Age differences in routine formation: the role of automatization, motivation, and executive functions

**DOI:** 10.3389/fpsyg.2023.1140366

**Published:** 2023-07-06

**Authors:** Irene van de Vijver, Lotte P. Brinkhof, Sanne de Wit

**Affiliations:** ^1^Habit Lab, Department of Clinical Psychology, University of Amsterdam, Amsterdam, Netherlands; ^2^Amsterdam Brain and Cognition, University of Amsterdam, Amsterdam, Netherlands; ^3^Department of Experimental Psychology, Helmholtz Institute, Utrecht University, Utrecht, Netherlands

**Keywords:** medication adherence, routine formation, aging, automatization, motivation, prospective memory, habit

## Abstract

Medication adherence can be vital for one’s health, especially in older adults. However, previous research has demonstrated that medication adherence is negatively affected by age-related cognitive decline. In the current study we investigated whether older adults are able to compensate for this decline by relying more on the formation of efficient, automatized routines. To this end, we directly compared daily (placebo) medication adherence in a healthy sample of 68 younger (18–29  years) and 63 older adults (65–86  years) over a period of 4  weeks. We show that despite an age-related decline in cognitive functions (i.e., poorer working memory, prospective memory, task switching, and goal-directed control), older adults adhered better to a daily pill intake routine than younger adults did and, in line with our hypothesis about increased routine formation, reported higher subjective automaticity of pill intake. Across age groups, automatization of pill intake was related to intake regularity and conscientiousness, but not to individual differences in habit tendency as measured in the lab nor to explicit strategic planning. Crucially, the age-related increase in pill intake adherence was mediated by experienced automatization as well as motivation. These findings demonstrate that intact habitual processes and high motivation aid older adults in successfully forming daily routines.

## 1. Introduction

Many health-related actions in daily life, such as medication intake, need to be repeated on a regular basis. Such regularly repeating behaviors can be vital for long-term health but often prove difficult to sustain, potentially because they are aimed at long-term goals and lack immediate gratification (DiMatteo, 2004; Briesacher et al., 2008). The formation of efficient, automatized routines may therefore be crucial to maintain these behaviors (Wood and Neal, 2016). This may be especially the case for older adults. Successful medication adherence depends, at least initially, on multiple cognitive control functions. For example, one needs to remember to take a pill at a certain moment in time (prospective memory; PM), to keep this intention as well as the relevant action online until the moment of execution (working memory; WM), and to switch from the task at hand to the action of taking the pill (switching). Indeed, medication adherence is negatively affected by age-related declines in PM (Zogg et al., 2012; Woods et al., 2014; Ihle et al., 2017), executive control functions including task switching, and working memory (WM; Insel et al., 2006; Smith et al., 2017). Nonetheless, medication adherence does not seem to decrease with increasing age perse (Park et al., 1999; Rolnick et al., 2013; Uchmanowicz et al., 2019), suggesting that older adults are able to compensate for these cognitive declines. Indeed, whereas performance on PM tasks (tasks that require one to remember to perform an action in the future) in the lab is usually worse in older than in younger adults, performance of real-life PM tasks is often better in older adults (Bailey et al., 2010; Mattli et al., 2014; Hering et al., 2016; Schnitzspahn et al., 2016). This differential impact of aging on performance on PM tasks in the lab and in daily life is also referred to as the ‘age-prospective memory paradox’ (Rendell and Craik, 2000). We suggest that one way in which older adults may achieve this better performance in real life is by relying more on automatic or habitual processes when forming real-life routines. To investigate this possibility, the present study determined whether older adults automatize a (placebo) medication intake routine faster than younger adults. Furthermore, we investigated how this is affected by individual differences in cognitive functions versus factors related to habit formation. Although overall medication adherence may not be affected in healthy aging, medication non-adherence is a particularly important issue in older adults. Their use of prescription medicines is higher than in other age groups (Barnett et al., 2012; Mielke et al., 2020), while non-adherence can result in more negative health outcomes (Hughes, 2004; Walsh et al., 2019). Therefore, insight into the roles of cognitive and automatic processes in the successful formation of a daily routine is highly relevant, and may inform interventions to support medication adherence.

A behavior can become automatic, or habitual, when the action is repeatedly performed in the same context. According to popular accounts, this may be due to the formation of a direct stimulus–response (S-R) association, enabling the context to become the stimulus (S) that automatically triggers the related behavior (R) (de Wit and Dickinson, 2009). Because habitual actions are automatically triggered by internal or external stimuli, they are less cognitively demanding and faster than goal-directed actions, albeit also less flexible (Wood and Rünger, 2016). Thus, increasing the automaticity of a regularly repeating action may benefit the continued execution of the intended action. In a previous study in young adults we indeed demonstrated that individual differences in the experienced automaticity of a novel, daily pill intake routine, as measured with the Self-Report behavioral Automaticity Index (SRBAI, Gardner et al., 2012), were positively related to pill intake adherence (van de Vijver et al., 2023). Furthermore, habit strength is an important predictor of real-life medication adherence (Alison Phillips et al., 2013; Fernandez-Lazaro et al., 2019; Badawy et al., 2020). So far, the effect of healthy aging on the automatization of a routine has not been directly investigated, but the age-related decline in remembering to perform an intended action in the future (i.e., PM) does seem to be the smallest when the intended actions are cue-triggered and regularly repeating, requiring little cognitive monitoring (Rose et al., 2010; Kliegel et al., 2016; Zuber and Kliegel, 2020). Relatedly, experimental research suggests that older adults rely more on habitual (as opposed to goal-directed) control when learning new behaviors than younger adults (de Wit et al., 2014). We therefore hypothesized that older adults not only automatize a new routine faster than younger adults, but are also more dependent on this process of automatizing such a regular task than younger adults, in whom executive control functions and WM are relatively strong.

There are several factors that may play a role in routine formation and that may affect age-related differences, including the regularity of the behavior. When a regularly repeating action is performed in the same context, the context or stimulus that becomes associated with the to-be-repeated action can be purely physical, such as the bathroom, but it can also constitute an event, such as brushing your teeth. This suggests that people who adhere to a more regular pattern when performing a repeating action also experience a more consistent and recurring context for this behavior, enabling them to more easily implement a new routine in their daily schedule. Therefore, we hypothesized that the temporal regularity of the pill intake action would be positively related with automatization of this routine. Similarly, and in line with recent findings in a study in younger adults, we also expected a positive relation between general lifestyle regularity and automaticity of a new pill intake routine (van de Vijver et al., 2023). Based on the same study we predicted that a higher tendency to rely on habits – as measured with an outcome-revaluation task in the lab – and higher levels of conscientiousness would positively predict automatization of pill intake as well (van de Vijver et al., 2023). Crucially, older adults generally show a higher lifestyle regularity than younger adults (Monk et al., 1997, 2002), demonstrate a stronger habit tendency as measured in the lab (de Wit et al., 2014), and have higher levels of conscientiousness than younger adults (Jackson et al., 2009; Specht et al., 2011). Therefore, we hypothesized that this would allow older adults to automatize routines faster than younger adults, thereby supporting their medication adherence.

Another related factor that may aid the automatization of a regularly repeating behavior, is a strategic planning strategy known as ‘implementation intentions’. Specifically, rather than specifying the goal of an action, such as ‘I want to take my medication every day’, implementation intentions directly link the intended behavior to a pre-existing cue using an ‘if … then …’ formulation, such as ‘If I finish breakfast, then I will take my medication’ (Gollwitzer, 1999). Implementation intentions have been demonstrated to increase the automatization of a novel routine (Orbell and Verplanken, 2010), and to support goal pursuit (Hagger et al., 2016). Importantly, implementation intentions can be used to improve older adults’ performance of various real-life health behaviors (Liu and Park, 2004; Ziegelmann et al., 2007; Brom and Kliegel, 2014). In the current study, we investigated the effect of strategic planning on automatization of a daily pill intake routine by randomly allocating half of the participants to a goal-intention and the other half to an implementation-intention condition. We hypothesized that participants who used implementation as compared to goal intentions would take their pills more regularly and automatically, and as a result show higher pill intake.

To investigate these hypotheses, younger (18–30 years) and older participants (≥65 years) were asked to take a placebo pill every day for 4 weeks. To objectively measure pill intake, pills were provided in containers that registered the time and date of every opening. Participants reported their experienced automaticity of pill intake on a weekly basis by answering an online questionnaire that included the SRBAI. Before the onset of pill intake, half of the participants formulated an implementation intention, and the other half a goal intention. During two lab visits before and one visit after the pill-taking phase, multiple cognitive and personality measures were obtained. We expected that older adults would show age-related declines in cognitive control functions that are relevant for successful medication adherence (i.e., PM, WM, switching), which were tested by including a computerized measure of PM, the Operation Span test (to assess WM; Turner and Engle, 1989), and a computerized task-switching task. We also predicted that older adults would rely more on automatic processes when forming a novel pill intake routine, as reflected in higher subjective automaticity. Furthermore, we expected that routine automatization would be positively affected by variables that have previously been demonstrated to be relevant to habit formation (i.e., regularity, habit tendency, conscientiousness, strategic planning; van de Vijver et al., 2023), thereby indirectly supporting daily adherence. Specifically, the Symmetrical Outcome-Revaluation Task (SORT) was included to test whether a higher tendency to rely on habits in the lab was related to routine formation in daily life.

## 2. Methods

### 2.1. Participants

Seventy-one younger (18–30 year) and 74 older (≥65 year), community-dwelling adults participated in this study. Before participation, a screening interview was administered via phone (older adults) or internet (younger adults). Exclusion criteria were a diagnosed neurological or psychiatric disorder, a history of brain damage, excessive alcohol or drug consumption, the use of psychotropic medication, and visual, auditory, or motor problems that could affect test performance. For the older adults, the interview also comprised the *Cognitieve Screeningstest 20* (Cognitive Screening Test 20, CST-20; Deelman et al., 1989). Older adults with a score below 17, suggesting more severe cognitive decline than can be expected with healthy aging (Van Toutert et al., 2016), were also excluded. Participants received a reward of € 80 or course credits for participation. This study was approved by the Ethics Review Board of the Faculty of Social and Behavioral Sciences of the University of Amsterdam.

After participation, the data of three younger and 11 older participants had to be excluded because they (1) refrained from further participation after the first lab session (2 older), (2) had incomplete or unreliable pill data (e.g., long holiday in between; 3 younger and 3 older), or (3) did not have an SRBAI score on day 28 (see section 2.2, 6 older). The data of the remaining 68 younger (22 male; 18–29 years, M 21.4, SD 2.67) and 63 older (18 male; 65–86 years, M 71.4, SD 5.45) participants are included in all reported measures and analyses, unless otherwise specified. Although no specific measure of current educational engagement was obtained, the large majority of the included younger adults were attending a university or university of applied sciences when participating in the study, while 38 of the 63 included older adults (~60%) reported to have obtained a degree from a university or university of applied sciences.

### 2.2. Procedure

#### 2.2.1. General study procedure

**Figure 1:**
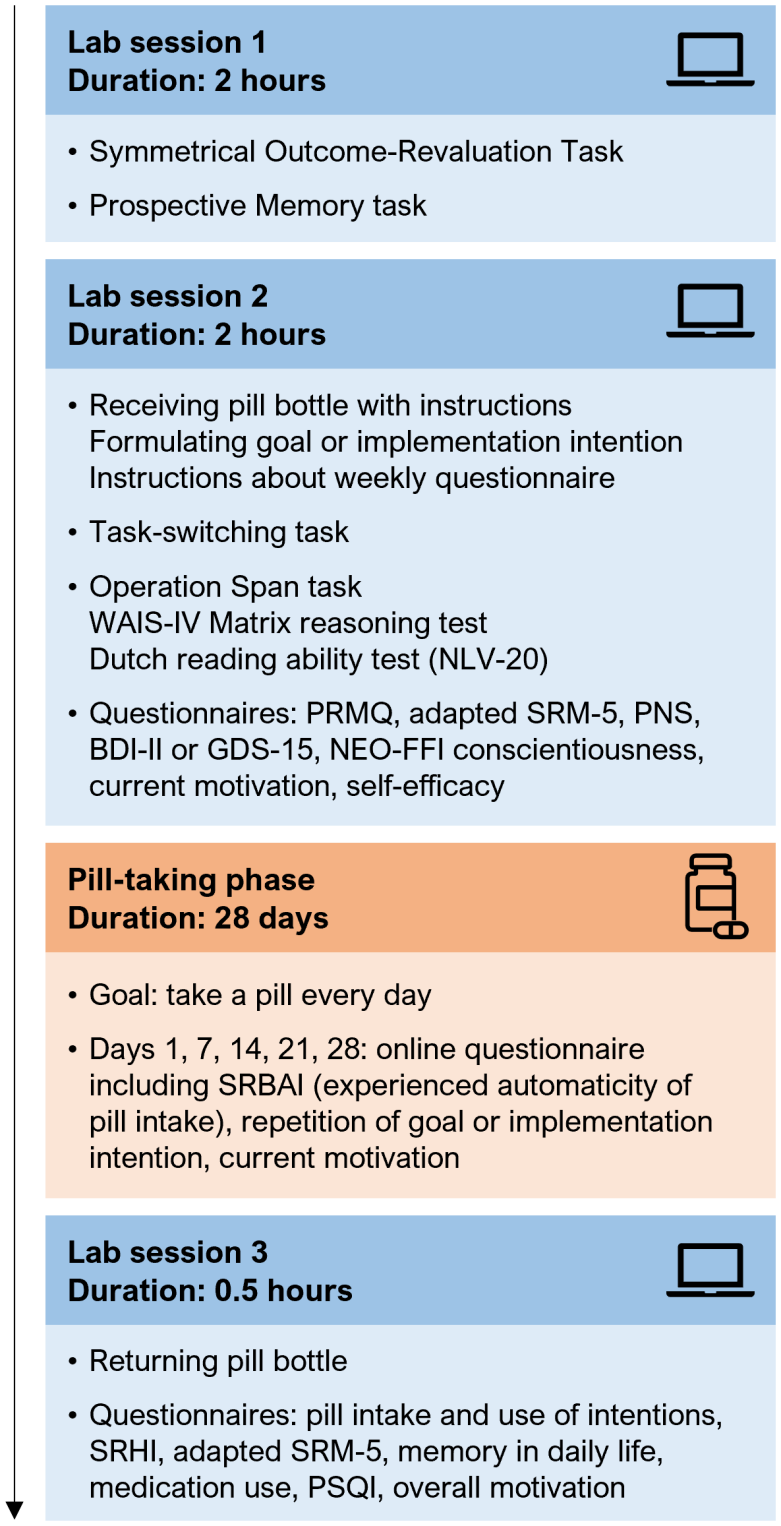
Overview of the study procedure. Participation consisted of three lab sessions, separated by a pill-taking phase of 4  weeks. The presentation order of the Symmetrical Outcome-Revaluation Task and Prospective Memory task (session 1), and of the Operation Span task, WAIS-IV Matrix reasoning test, and Dutch reading ability test (session 2) was randomized over participants. All computer tasks, neuropsychological tests, and questionnaires are described in detail in section 2.3 and the Supplementary methods. WAIS-IV, Wechsler Adult Intelligence Scale-IV; NLV, Nederlandse Leesvaardigheidstest voor Volwassenen (Dutch Reading Ability Test); PRMQ, Prospective and Retrospective Memory Questionnaire; SRM-5, Social Rhythm Metric Short Form; PNS, Personal Need for Structure scale; BDI-II, Beck Depression Inventory-II; GDS-15, Geriatric Depression Scale-15; NEO-FFI, Neuroticism-Extraversion-Openness Five Factor Inventory; SRBAI, Self-Report Behavioural Automaticity Index; SRHI, Self-Report Habit Index; PSQI, Pittsburg Sleep Quality Index.

Participation started with two lab sessions, which were scheduled between 1 and 12 days apart (M 2.85 days, SD 2.25). During both sessions, participants performed computer tasks and neuropsychological tests, and filled out questionnaires (see Figure 1). During the second lab session, participants were asked to take a placebo pill on a daily basis for 28 days, starting the next day. On days 1, 7, 14, 21, and 28 of this pill-taking phase, participants received a text message and email with a link to a questionnaire about their current pill intake experience. A reminder was sent 1 day later if the questionnaire was not filled out yet. A third lab visit was scheduled on or after the last day of the pill-taking phase, with a maximum of 18 days later (M 3.98, SD 2.81). For an overview of the complete study procedure, see Figure 1. The questionnaires that were used during the screening, lab sessions, and pill-taking phase were all administered using Qualtrics,^1^ and weekly messages and reminders were sent using the in-house Lotus web tool.^2^

#### 2.2.2. Formulation of goal and implementation intentions

At the start of the second lab session, participants received the pill bottle and instructions about its use. They were told they could take the bottle with them when they were away from home. At the societal level, participants were informed about the risks of medication non-adherence in older adults, and about forgetting as an important contributor to non-adherence. They were explained that the current study aimed to contribute to decreasing medication non-adherence by mapping which cognitive abilities and personality traits could predict successfully taking medication, to allow for more individually tailored assistance. The experimenter explained that it was very important for both age groups to really try and take a pill every day, because only in that way the study would produce meaningful results. At a personal level the participants were encouraged to come up with a strong and relevant motivation for themselves to make sure they would do their best, and were suggested to think of an older relative or neighbor that might struggle with medication intake now or in the future.

After participants came up with a relevant personal motivation, they continued with formulating an implementation or goal intention to support pill intake. After the societal and personal relevance of the study were emphasized, participants formulated an implementation or goal intention to support pill intake. The goal intention was ‘I will take a pill every day!’. Implementation intentions were formulated as an ‘if [cue], then I will take the pill!’ statement. Participants identified a personal cue, which had to be a daily behavior or situation, such as putting on their glasses in the morning or having breakfast. The cue could not be a time and could not be related to their normal medication use. Once an appropriate cue was decided on, the participant was asked to visualize the complete cue-pill intake sequence and consider whether they foresaw any obstacles to using this event as the cue (Scullin et al., 2017). In both intention conditions participants had to write down their intention three times, read it out loud five times, and repeat it three times from the top of their head. They were instructed that it was not allowed to use external aids to help them remember to take the pill. Finally, participants received instructions about the weekly questionnaires that they would receive during the pill-taking phase.

### 2.3. Materials

#### 2.3.1. Pill bottles with medication event monitoring system

Participants received 35 placebo pills in a white, plastic, non-transparent container with a white MEMS® TrackCap (Medication Event Monitoring System, AARDEX group;^3^ together referred to as the pill bottle). The micro-circuitry in this type of cap records the date and time each time the cap is removed from the container. The container and cap did not provide any information about the content, or about previous or required openings. Pills were obtained from a local pharmacy. Participants were informed that the pills were placebo.

After participants returned their pill bottle, intake data were exported from www.aardexgroup.com and automatically registered times and dates of bottle openings were corrected. Specifically, bottle openings after midnight (0.00–4:00) were considered to be part of the previous day (i.e., 24:00–28:00). Bottle openings between 4.00 and 5.00 were considered part of the next day if participants showed a pattern of getting up early, and considered part of the previous day (28:00–29:00) if this was not the case. Based on these dates and times we calculated the number of pills that were taken each week, and during the complete pill-taking phase. If participants opened the pill box more than once on the same day, the time of the first opening was used as time of intake (this occurred on 1/2/3/4 day(s) in 10/3/3/3 younger and 7/2/3/0 older participants, respectively). Such commission errors are thought to result from a problem with retrospective rather than prospective memory (Ihle et al., 2017). Because they therefore seem to be unrelated to the cognitive mechanisms that are examined in the current study, and because the action of taking a pill is being performed on days on which the bottle is opened more than once and these days can therefore contribute to increasing automaticity and building a routine, commission errors are not considered errors in the current study. As a measure of intake (ir)regularity, we calculated the standard deviation of the registered intake times.

#### 2.3.2. Self-report habit index and self-report behavioral automaticity index

The 12-item Self-Report Habit Index (Verplanken and Orbell, 2003) assesses multiple aspects of habit strength, including experienced automaticity, the behavioral repetition history, the experienced difficulty controlling the behavior, and the relation with personal identity. The current version was administered in the third lab session, and we specified that all questions referred to the complete pill-taking phase. All items started with ‘Taking the pill was something…’ followed by the specific statement of that item. Participants used virtual sliders to indicate to what extent they agreed with each statement, with scores ranging from ‘strongly disagree’ (0) to ‘strongly agree’ (100). The scores of the 12 items were averaged into one SRHI score.

The Self-Report Brief Automaticity Index (Gardner et al., 2012) only includes the four questions from the SRHI that focus on the experienced automaticity of a behavior, in this case taking the pill: ‘Taking the pill was something I did automatically’, ‘Taking the pill was something I did without having to consciously remember’, ‘Taking the pill was something I did without thinking’, and ‘Taking the pill was something I did before I realized I was doing it’. When the SRBAI was filled out on day 1 (after the first pill was taken), it referred to pill intake ‘so far’, while on the other days (7, 14, 21, and 28) it referred to pill intake ‘in the last week’. Participants used the same 0–100 answering scales. The scores on the four items were averaged per administration of the SRBAI. Scores were considered missing when the SRBAI was completed too early, more than 2 days too late, or not at all. Participants missing the final measurement (at day 28) were excluded from all further analyses (resulting in *N* = 131 for most analyses). Participants missing the first measurement (4 younger and 3 older participants) were only excluded from the analyses of the development of automaticity over time (*N* = 124). Missing values in subsequent weeks (at day 7, 14 and 21) were imputated for this analysis using linear interpolation (2 younger and 2 older participants missed one measurement).

#### 2.3.3. Static symmetrical outcome-revaluation task

**Figure 2:**
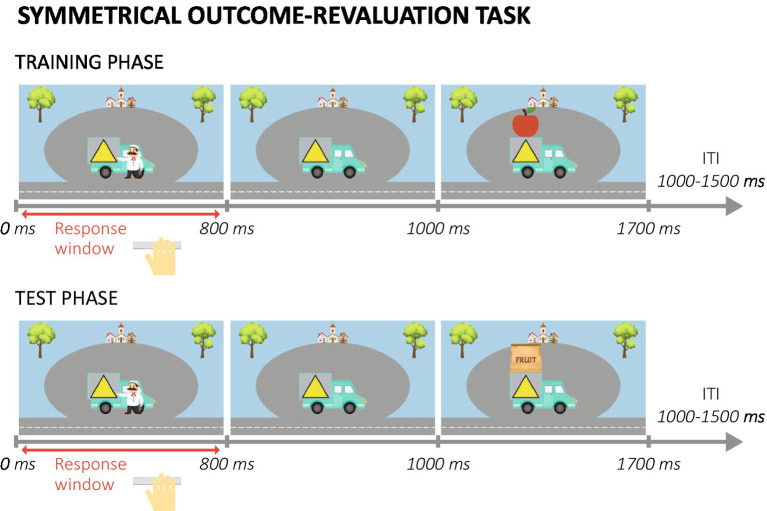
Example trials in the training and test phase of the symmetrical outcome-revaluation task. ITI, inter-trial interval.

This outcome-revaluation paradigm (based on Watson et al., 2022) consisted of two main phases (for a detailed description of the task procedures see Supplementary methods S1.1). In the training phase, participants were presented with trucks with a colored symbol superimposed (stimulus; see Figure 2). The symbol indicated which type of fruit (outcome, O) could be obtained from each truck. Before each block, participants were informed which two fruits were valuable (go condition) and which two were not (no-go condition). Participants were instructed to only collect the valuable fruits, by pressing for the associated trucks. On each trial, after the response window finished the outcome fruit was presented above the truck. The same truck-fruit combinations remained valuable throughout the training. Thus, participants always had to press (Go response, R) for the same trucks (S), which should lead to a direct S-R association being formed. After the training phase, participants were asked to indicate their explicit knowledge about the stimulus-outcome relations in the task.

During the test phase, participants were still supposed to only collect valuable fruits by pressing for the associated trucks. However, outcome fruits were no longer presented, so participants had to rely on their previously learned truck-fruit (S-O) associations. In each test block a different combination of outcome fruits was valuable. Because specific truck-fruit combinations were always (non)valuable during training, the combination of the values during training and test resulted in two truck-fruit combinations being still valuable, two still not-valuable, two devalued, and two upvalued. This combination of value-congruent (still-valuable and still-not-valuable) and value-incongruent (devalued and upvalued) trial types allowed us to investigate the ability to flexibly adjust responses to stimuli when outcome value changed.

The test phase was followed by a second, baseline test, in which in each block four stimuli instead of four outcomes were devalued.

Three participants (all old) had no (complete) data and were therefore excluded from all SORT analyses (*N* = 128). The difference in performance accuracy between value-congruent and value-incongruent trials was used as measure of habit tendency.

#### 2.3.4. Prospective memory task

**Figure 3:**
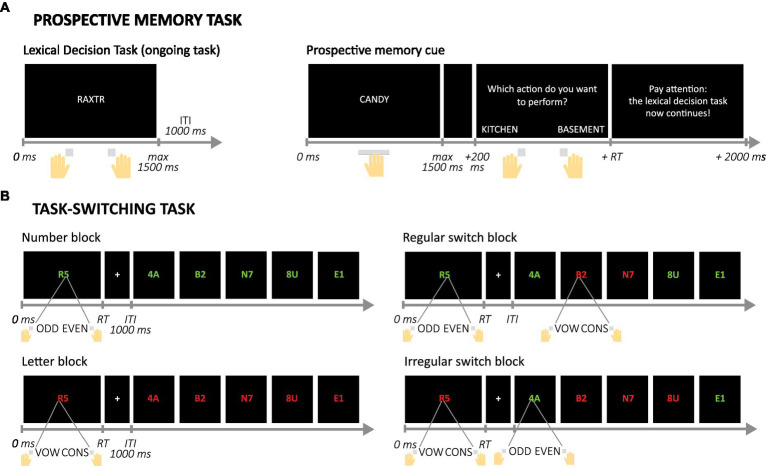
Example trials and trial sequences in the Prospective memory and Task-switching computer tasks. **(A)** Example trial in the ongoing, Lexical Decision Task and trial in which a prospective memory cue is presented and responded to. **(B)** Set of example trials in the four possible block types in the Task-switching task. Note that inter-trial intervals (ITI) are not displayed here after the second trial, but were presented in the actual task. Mappings between colors and assignments and between answers and buttons were counterbalanced over participants. RT, reaction time; vox, vowel; cons, consonant.

Participants performed a lexical decision task (LDT) with additional PM assignments (similar to Ihle et al., 2018). On each trial of the LDT, they had to indicate with a key press whether a 5-letter string was a word or non-word (see Figure 3A; Supplementary methods S1.2 for a detailed description of the task). Half of the trials featured words, the other half non-words. The task started with a practice block (30 trials) and a first test block consisting only of the LDT (108 trials). In the second and third test block, two cue words were presented six times each, intermixed with the 108 LDT trials (total of 120 trials per block). The cue words were associated with specific action words that the participants were instructed on before the block started. If participants saw a cue word they had to press the space bar and indicate the action related to the cue, after which the next LDT trial started. Participants received extensive instructions about how to respond to the PM cues before the second and third block, but no specific practice of this element of the task.

Eight participants (four younger) had no (complete) data for the PM task and one younger adult had a very low accuracy score in the first test block (LDT only) and was considered an outlier. Their data were therefore not included in the analyses of PM behavior (*N* = 122). The number of correctly detected PM cues (averaged over the two PM blocks) was used as a proxy of PM performance in the lab to relate performance on this task to pill intake and other measures.

#### 2.3.5. Task-switching task

On each trial participants saw a letter and a number (adapted from Sohn et al., 2000; see Figure 3B; Supplementary methods S1.3 for a detailed description of the task). Depending on the color of the symbols, the participant had to indicate either whether the digit was odd or even, or whether the letter was a vowel or a consonant. The task contained four types of blocks. In the number and letter blocks the color of the symbols was constant so participants only had to focus on either the numbers or the letters. In the regular switch blocks, the color switched every two trials, so switches were predictable and would allow for proactive control. In the irregular switch blocks, the order of the colors was unpredictable and the participant could only use the color on each trial to know the assignment, and had to rely on reactive control in these blocks. These two types of blocks were included to explore whether age-related differences in switching depended on the type of control that was required (Braver et al., 2005; Bugg, 2013; Manard et al., 2014). Participants were notified before each block of the upcoming block type.

For all analyses, the first trial of a block was disregarded, since this could never be a repetition or switch. The switch cost was defined as the average of the difference in RT between stay trials (same rule had to be applied) and switch trials (switch from number to letter rule or vice versa) in the regular and irregular switch blocks. Six participants (one younger) had no (complete) data for the task-switching task and were excluded from all related analyses (*N* = 125).

#### 2.3.6. Neuropsychological tests

WM was assessed with the Operation Span test (Turner and Engle, 1989). In this test, the participant was required to keep an increasingly long string of words in mind while performing simple mathematical operations. In the current version of the test (based on Oswald et al., 2015), the number of words per set varied between three and five. Each set type was presented three times, resulting in a total of nine sets. Performance was scored using the partial credit scoring system (Conway et al., 2005). Fluid and crystallized intelligence were assessed with the Matrix reasoning subtest of the Wechsler Adult Intelligence Scale-IV (WAIS-IV; Wechsler, 2008) and the Dutch Reading Ability Test (Nederlandse Leesvaardigheidstest voor Volwassenen, NLV; Schmand et al., 1991), respectively.

#### 2.3.7. Questionnaires

Questionnaires that were administered in the second lab session included the Prospective and Retrospective Memory Questionnaire (PRMQ; Smith et al., 2000), an adapted version of the Social Rhythm Metric short form to assess lifestyle regularity (SRM-5; Monk et al., 2002), the Personal Need for Structure scale (PNS, Neuberg and Newsom, 1993), for the younger adults the Beck Depression Inventory II (BDI-II; Beck et al., 1961, 1996; van der Does, 2002) and for the older adults the Geriatric Depression Scale-15 (GDS-15; Yesavage et al., 1982; Sheikh et al., 1986; Burke et al., 1991), the conscientiousness scale of the Neuroticism-Extraversion-Openness Five Factor Inventory (NEO-FFI; Costa and McCrae, 1989), as well as questions about the participants’ motivation and self-efficacy regarding pill intake during the next 4 weeks. One older participant did not complete these questionnaires and was excluded from all related analyses.

The questionnaire that had to be filled out on a weekly basis during the pill-taking phase included the SRBAI (Gardner et al., 2012) as well as questions asking the participant to repeat their intention and to indicate their current motivation to take the pill every day. Seven participants (4 younger and 3 older) did not fill out the first measurement (at day 1) and were excluded from the analyses of the development of motivation over time. Missing values in subsequent weeks (at day 7, 14 and 21) were imputated using linear interpolation (note that we used the same missing-value criteria as for the SRBAI).

During the third lab session, participants filled out questionnaires comprising (1) a section about pill intake, including questions about the use of the formulated intention and experience thereof, the SRHI (Verplanken and Orbell, 2003), and questions about time spent away from home, (2) again the adapted version of the SRM-5 (Monk et al., 2002), (3) questions about subjective memory experiences in daily life, (4) questions about regular medication use, (5) the Pittsburg Sleep Quality Index (PSQI; Buysse et al., 1989), and (6) questions about motivation. One younger participants did not complete the first four questionnaires, another younger participant did not complete questionnaire five and six. Data of the adapted SRM-5 and PSQI have not been included in the analyses because response patterns on both measures deviated too much from common findings in healthy adults, possibly because of misinterpretation of questions by participants.

### 2.4. Statistical analyses

Pill intake data and data from the computer tasks were processed in Matlab using custom-written scripts. Statistical analyses were carried out in R 3.5.1 (R Core Team, 2018) with alpha criteria of 0.05.

Several two-way analyses of variance (ANOVAs), with Age group (young, old) and Intention condition (II, GI) included as predictors, were conducted to examine whether both factors contributed to individual differences in the scores on the neuropsychological tests, and questionnaires prior to the start of the pill-taking phase (see Table 1). If there was no significant Age group × Intention condition interaction effect, a type II sum of squares (SS) approach was more powerful (following the principle of marginals) as compared to type III (Langsrud, 2003). Therefore, we tested for interaction first and continued with the analysis for main effects when no significant results were found. Otherwise, a type III SS approach was used. A similar approach was used for the two-way ANOVAs adopted to evaluate differences in pill intake regularity (during the study phase), as well as motivation at the end. In case of the BDI and GDS, two-tailed *t*-tests were used to assess the Intention condition effect in the two separate age groups.

**Table 1:** Demographics of participants in different age and intention groups; note that for the PRMQ a higher score indicates worse prospective or retrospective memory.

		Younger		Older	
	Scale	GI	II	GI	II
Males: Females		08:26	14:20	07:24	11:23
Age		21.7 (2.91)	21.2 (2.43)	71.0 (4.91)	71.7 (5.96)
O-Span*	0–100%	62.6 (16.0)	70.1 (18.3)	51.2 (17.2)	51.2 (16.1)
WAIS-IV MR - Raw*	0–26	20.7 (3.44)	21.0 (3.95)	15.5 (3.62)	16.1 (3.94)
WAIS-IV MR - Norm	1–19	11.6 (2.96)	11.6 (2.97)	11.2 (2.07)	11.7 (2.11)
NLV*	0–100	86.8 (5.14)	86.4 (5.55)	93.3 (5.30)	92.9 (6.20)
Prospective memory (PRMQ)*	8–40	14.8 (3.25)	14.8 (4.28)	13.0 (2.30)	13.2 (2.28)
Retrospective memory (PRMQ)	8–40	13.1 (3.38)	13.0 (2.60)	13.2 (2.51)	13.8 (2.60)
Personal need for structure	12–84	48.5 (9.92)	46.4 (11.4)	49.8 (14.6)	47.6 (9.78)
Conscientiousness	12–60	44.5 (5.65)	43.0 (6.94)	46.1 (5.44)	44.3 (5.44)
Depressive symptoms: BDI	0–63	5.67 (4.00)	6.30 (6.52)	–	–
Depressive symptoms: GDS-15	0–15	–	–	1.23 (1.81)	1.03 (1.51)
Initial motivation to take pill daily	0–7	6.38 (0.60)	6.21 (0.69)	6.37 (1.19)	6.58 (0.56)
Self-efficacy to take pill daily**	0–7	5.88 (0.98)	6.24 (0.78)	6.37 (0.76)	6.58 (0.50)

GI, goal intention, II, implementation intention, MR, matrix reasoning; all values M (SD), *significant difference between age groups, **significant difference between age groups and significant difference between intention conditions.

To investigate the effect of Age group, Intention condition, and Week (1–4) on pill intake, self-reported automaticity, and motivation during the study period, we employed mixed-design ANOVAs, as provided by the R software package ‘afex’ (Singmann et al., 2021). The ANOVAs were complemented with two-tailed *t*-tests or, in case of unequal variances among subgroups, Welch’s *t*-tests. Again, a type II SS approach was only used when no interactions were observed (default: type III SS). All *p*-values involving repeated-measures factors were corrected for violations of sphericity. The appropriate correction in case of violations of sphericity was based on the Greenhouse–Geisser estimate of sphericity (ξ): the Greenhouse–Geisser correction was used when ξ < 0.75, the Huynh-Feldt when ξ > 0.75. Associations between categorical variables were assessed using Chi-square tests of independence. When appropriate, *post-hoc z*-tests on the residuals were used to identify the exact associations. For the *z*-tests, *p*-values reflect the significance compared to a pre-determined criterion value rather than an exact statistic.

To predict pill intake using other study variables (e.g., SRBAI and O-Span scores), multiple logistic regression models were used. Because we observed a ceiling effect for the total number of pills that was taken (28), two pill intake groups were created (Low: <27 pills; High: ≥27 pills) and Pill intake was considered a binary variable for all regression analyses. Continuous independent variables were mean centered. To control for differences in the distribution of younger and older participants over the pill groups (see Results section 3.4), the factor age group was included as a covariate when the independent variable differed significantly between the groups. If a (marginally) significant interaction between the independent variable and age group existed, we focused on the independent variable x group interaction term, otherwise the output of the model without the interaction is reported. Contrary to the age groups, the intention conditions (II/GI) were equally distributed over the pill intake groups, *χ*^2^(1) = 0.604, *p* = 0.437, *V* = 0.053. Outliers were examined and removed (if necessary) using studentized residuals (Zhang, 2016); all continuous predictors were mean centered prior to model estimations. The contribution of each term in the models was evaluated by using the Wald Chi-squared test.

To predict the continuous SRBAI scores using other relevant study variables (e.g., pill intake regularity, conscientiousness scores, and O-Span), linear models were used. Again, continuous independent variables were mean centered. We followed the same reasoning to in-or exclude the factors Age group (young/old) or Intention condition (GI/II) as covariate in these models as described for the multiple logistic regression models. However, when theoretically relevant, the factor Intention condition was also included as covariate and the output of the model including the independent variable x Intention condition interaction term was used. Outliers were removed using cooks distance, using the traditional 4/n criterion (Cook, 1977; Cook and Weisberg, 1982).

Multiple mixed-design ANOVAs, again complemented with t-tests, were performed to analyse the effects of age group and task conditions on behavior in the static Symmetrical Outcome-Revaluation task, the Prospective Memory task, and the Task-switching task. The specific factors included per ANOVA are detailed in the Supplementary results. Habit tendency and switch cost were also used as independent variables to predict SRBAI scores and pill intake, respectively, with logistic and linear regression models in accordance with previously described approaches, respectively. The number of correctly detected PM cues in the PM task was compared among pill groups for the two different age groups separately using two *t*-tests.

## 3. Results

### 3.1. Age differences in neuropsychological tests and questionnaire scores

Descriptives, scores on questionnaires and cognitive tests, and the distribution of the included participants over intention conditions are presented in Table 1. Age-related differences in WM, fluid intelligence, and crystallized intelligence were in line with commonly observed effects of increasing age: older compared to younger adults had lower scores on the O-Span, *F*(1,127) = 26.55, *p* < 0.001, *η*_p_^2^ = 0.17, and WAIS-IV matrix reasoning subtest, *F*(1,127) = 59.16, *p* < 0.001, *η*_p_^2^ = 0.32. When matrix reasoning scores were converted using age-corrected norm scores they no longer differed between age groups, *F*(1,127) = 0.11, *p* = 0.742, *η*_p_^2^ = 0.0009, confirming the age-related effect on the difference in raw scores. Older adults achieved higher scores than younger adults on the NLV-20, *F*(1,127) = 44.57, *p* < 0.001, *η*_p_^2^ = 0.26. Scores on all three measures did not differ between intention conditions, all *p* > 0.19.

In line with previous literature (Kliegel et al., 2016; Zuber and Kliegel, 2020), self-reported PM was lower in older compared to younger adults, *F*(1,126) = 9.29, *p* = 0.003, *η*_p_^2^ = 0.07. In the current sample, older adults did not differ from younger adults in retrospective memory, *F*(1,126) = 0.69, *p* = 0.409, *η*_p_^2^ = 0.005, possibly because we did not include participants that showed signs of cognitive decline. There were also no differences between age groups in personal need for structure, *F*(1,126) = 0.37, *p* = 0.546, *η*_p_^2^ = 0.003, or conscientiousness, *F*(1,126) = 1.96, *p* = 0.164, *η*_p_^2^ = 0.015. Most participants did not show depressive symptoms, only 4 older and 5 younger adults showed signs of possible (GDS-15) or mild to moderate (BDI-II) depression. Again, intention conditions did not differ on these measures (all *p* > 0.12).

At the start of the pill-taking phase, motivation to take a pill on a daily basis did not differ between age groups or intention groups, all *p* > 0.16. However, older compared to younger adults did report a higher confidence in their ability to take a pill every day, *F*(1,127) = 9.13, *p* = 0.003, *η*_p_^2^ = 0.07, as did participants in the implementation compared to the goal intentions condition, *F*(1,127) = 4.37, *p* = 0.038, *η*_p_^2^ = 0.033 (no significant interaction, *F*(1,127) = 0.28, *p* = 0.598, *η*_p_^2^ = 0.002).

### 3.2. Pill intake behavior

**Figure 4:**
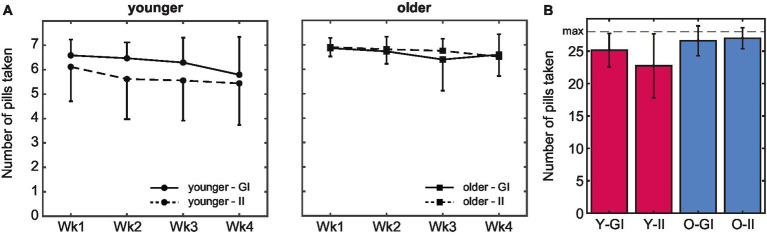
Pill intake behavior. **(A)** The average number of pills taken was higher in older compared to younger adults. In both age groups, intake peaked in the first week. **(B)** Whereas the total number of pills that was taken during the 4-week pill intake phase did not differ between intention conditions in older adults, intake was higher in the goal-intention compared to the implementation-intention condition in younger adults (error bars indicate standard deviations; Wk, week; Y, younger; O, older; GI, goal intentions; II, implementation intentions).

Both age groups managed to take a pill on most of the 28 days, with total intake ranging from 12–28 pills in younger and 19–28 pills in older adults. Fourteen younger and 38 older adults managed to take a pill on all days. The total number of pills that was taken was even higher in the older (M 26.8, SD 1.97) compared to the younger age group (M 23.9, SD 4.09), *F*(1,127) = 26.86, *p* < 0.001, *η*_p_^2^ = 0.17. Pill intake decreased over time, *F*(2.85, 362.38) = 8.19, *p* < 0.001, *η*_p_^2^ = 0.61 (Figure 4A): the number of pills that was taken was lower in the second compared to the first week, *t*(130) = 2.14, *p* = 0.034, *d* = 0.21, but did not decrease anymore thereafter (all *p* > 0.14). This decrease did not differ between age groups, *F*(2.85, 362.38) = 1.15, *p* = 0.327, *η*_p_^2^ = 0.06.

There was no main effect of intention condition, *F*(1,127) = 3.33, *p* = 0.071, *η*_p_^2^ = 0.03 (Figure 4B). A significant interaction effect of age group and intention condition, *F*(1,127) =, *p* = 0.012, *η*_p_^2^ = 0.05, indicated that pill intake did not differ between intention conditions in older adults, *t*(51.4) = −0.79, *p* = 0.435, *d* = 15.16, whereas intake was higher in the goal than the implementation intention condition in younger adults, *t*(49.8) = 2.52, *p* = 0.015, *d* = 7.36 (but see section 3.3 for spontaneous planning in the goal-intention condition). Importantly, in both intention conditions intake was higher in older (GI: M 26.6, SD 2.31, II: M 27.0, SD 1.62) than in younger adults (GI: M 25.1, SD 2.58, II: 22.7, SD 4.93), both *p* < 0.021.

### 3.3. Regularity of pill-taking behavior

Older adults not only took more pills than younger adults did, but also were more consistent in the time at which they took the pill, as was reflected by a lower standard deviation of intake times in older (M 0.06, SD 0.04) compared to younger adults (M 0.14, SD 0.07), *F*(1,127) = 62.58, *p* < 0.001, *η*_p_^2^ = 0.33. The more regular intake pattern of older adults matches their self-reported higher tendency to take the pill at a fixed moment each day compared to the younger adults (older: 73%, younger 52%), *χ*^2^(1) = 5.968, *p* = 0.015, V = 0.214.

There was a trend towards a higher regularity of pill-taking in the implementation compared to the goal intention condition (II: M 0.09, SD 0.06; GI: M 0.11, SD 0.07), *F*(1,127) = 3.68, *p* = 0.058, *η*_p_^2^ = 0.028, but no significant interaction of age and intention condition, *F*(1,127) = 0.24 *p* = 0.620, *η*_p_^2^ = 0.002. We had expected an effect of intention condition because participants in the implementation intention condition were proactively encouraged to associate pill intake with a specific daily behavior or event, and 100% of the older as well as the younger adults indeed reported afterwards to have done so. Interestingly, however, 57% of the older and 62% of the younger adults in the goal intention condition also reported to have spontaneously related pill intake to such a moment, and 63% of the older and 38% of the younger adults formulated a plan for this with an if-then structure. Thus, spontaneous planning regarding daily pill intake in the goal intention condition may have clouded any beneficial effects of the explicit implementation intentions.

The difference between age groups in pill intake and intake pattern may partially be explained by the fact that older adults were less likely than younger adults to be away from home at the usual moment that the pill was taken: 56% of the older adults and 81% of younger reported to be away from home at this moment on at least 1 day, *χ*^2^(1) = 8.93, *p* = 0.003, V = 0.27. In line with this finding, older adults also reported a lower estimated number of pills that was not taken due to absence (M 0.95, SD 1.68) compared to younger adults (M 3.02, SD 3.19), *F*(1,87) = 13.28, *p* < 0.001, *η*_p_^2^ = 0.13. This number did not differ between intention conditions, *F*(1,87) = 1.12, *p* = 0.292, *η*_p_^2^ = 0.01.

Another difference between age groups that may have affected their pill intake pattern, concerns their normal medication use. In line with the increase in prescribed medication with increasing age (Barnett et al., 2012; Mielke et al., 2020), older adults (51%) were more likely to take medication on a daily basis than younger adults (15%), *χ*^2^(1) = 19.10, *p* < 0.001, V = 0.38. While participants were not allowed to directly couple intake of the placebo pill to their normal medication intake, some participants (15.9% of all older and 4.5% of all younger participants) did report using the general rhythm of their regular medication use to facilitate intake of the placebo pill, for example by taking both pills in the morning. The number of participants that were prescribed medication did not differ between intention conditions, *χ*^2^(1) = 0.40, *p* = 0.529, V = 0.06.

To summarize, older adults adhered to a more regular intake schedule. Furthermore, there was a trend towards a more regular pattern of pill intake times with implementation compared to goal intentions. However, even though only participants in the implementation intention were specifically instructed to relate pill intake to a daily action or event, a significant part of the participants in the goal intention condition seems to have come up with a similar strategy by themselves, especially in the older group. Sticking to a regular intake pattern may have been less challenging for older adults because they were absent from home more less than younger adults. Furthermore, a small subgroup of the older participants may have been aided by their normal medication intake.

### 3.4. Experienced automaticity of pill-taking behavior

**Figure 5:**
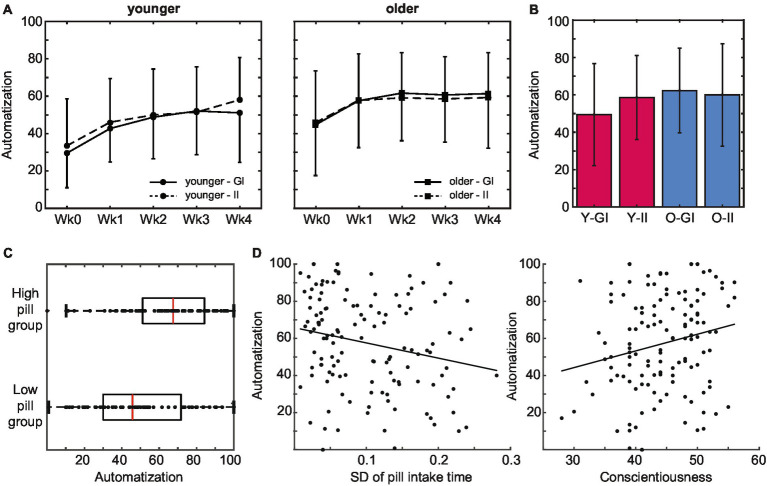
Subjective automatization of pill intake behavior. **(A)** Older adults reported a higher subjective automatization of pill intake than younger adults did. This difference was stable throughout the intake period. Automatization did not differ between intention conditions. **(B)** Experienced automatization at the end of the pill-taking phase (day 28) did not differ between age groups or intention conditions. **(C)** The level of automaticity that was reached at the end of the pill-taking phase positively predicted whether participants ended up in the low or the high pill group. **(D)** Subjective automatization was higher in participants that adhered to a more regular intake schedule as reflected in a lower SD of their pill intake times, as well as in participants with higher levels of conscientiousness (error bars represent standard deviations; Wk, week; GI, goal intentions; II, implementation intentions; Y, younger; O, older).

The experienced automaticity of pill taking increased over time: average SRBAI scores increased over weeks during the pill-taking phase, *F*(2.42, 289.7) = 35.81, *p* < 0.001, *η*_p_^2^ = 0.23 (see Figure 5A). Pair-wise comparisons indicated that this increase was significant between measurements on day 1 and day 7, *p* < 0.001, and between day 7 and day 14, *p* = 0.006, and remained stable afterwards, all *p* > 0.128. In line with our central hypothesis, older adults reported overall higher automaticity than younger adults did, *F*(1,120) = 7.59, *p* = 0.007, *η*_p_^2^ = 0.06. This difference did not change between weeks, *F*(2.41, 289.7) = 1.82, *p* = 0.156, *η*_p_^2^ = 0.015, suggesting that it emerged early in the routine formation process and remained stable. On the other hand, contrary to our expectations, strategic planning (i.e., intention condition) did not affect experienced automaticity over time (all p-values >0.64), nor did it influence the level of automaticity that was reached at the end of the 4 weeks of pill taking (all *p* > 0.115; Figure 5B).

During the final session in the lab, participants filled out the SRHI about their experiences during the complete pill-taking period. In line with the age difference in reported automaticity in the first 2 weeks, older adults (M 55.7, SD 20.2) experienced pill intake over the complete pill-taking period as more habitual than younger adults did (M 45.4, SD 18.0), *F*(1,126) = 9.44, *p* = 0.003, *η*_p_^2^ = 0.07. Similarly, when only the SRBAI questions were selected from the SRHI, pill taking was again experienced as more automatic by older (M 68.7, SD 17.9) compared to younger adults (M 58.1, SD 20.1), F(1,126) = 9.76, *p* = 0.002, *η*_p_^2^ = 0.07. There were no effects of intention condition on either measure (all *p* > 0.48).

Next, we investigated whether automaticity influenced pill intake. Because the number of pills that was taken was generally high and variability was limited, pill intake was converted into a categorical variable with a low intake group (intake below 27 pills) and a high intake group (intake at least 27 pills). In line with the age difference in pill intake, the majority of the younger adults (66%) emerged in the low pill group (*N* = 63; 45 younger, 18 older) whereas the majority of the older adults (71%) emerged in the high pill group (*N* = 68; 23 younger, 45 older), *χ*^2^(1) = 18.525, *p* < 0.001, *V* = 0.36. As predicted, the level of automaticity that was reached at the end of the pill-taking phase positively predicted whether participants ended up in the low or the high pill group, *χ*^2^(1) = 10.543, *p* = 0.001 (Figure 5C). Indeed, SRBAI scores at day 28 were higher in the high intake (M 65.2, SD 23.4) than in the low intake group (M 48.9, SD 24.7), *t*(129) = −3.88, *p* < 0.001, *d* = 0.68.

We also investigated whether automaticity was related to regularity and conscientiousness (Figure 5D). The predicted relationship between the regularity of the intake pattern and the automaticity at the end of the pill-taking phase was indeed significant, *B* = −99.58, *p* = 0.012. Participants that adhered to a more regular intake schedule (i.e., lower SD of intake) reported a higher experienced automaticity. Finally, automaticity at the end of pill taking was also positively predicted by individual differences in conscientiousness, *B* = 1.36, *SE* = 0.37, *r* = 0.32, *p* < 0.001.

To sum up, the experienced automaticity of pill intake increased over time. Automatization was faster in older than in younger adults. In line with our hypotheses, the increase in automatization was positively related to the number of pills that were taken, as well as to the regularity of the intake pattern and conscientiousness.

### 3.5. Motivation to take the pills on a daily basis

**Figure 6:**
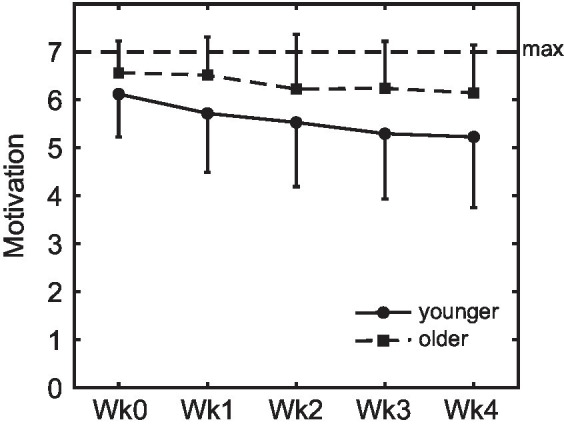
Motivation to take the pills on a daily basis. Motivation was higher in older compared to younger adults, and decreased over weeks in both groups (error bars represent standard deviations; Wk, week).

Whereas there were no age differences in initial motivation to take a pill daily as measured during the second lab session before the pill-taking phase (see section 3.1), motivation during the pill-taking phase was higher in older (M 6.35, SD 0.94) than younger adults (5.55, SD 1.32, Figure 6), *F*(1, 120) = 24.06, *p* < 0.001, *η*_p_^2^ = 0.17. Motivation decreased over weeks in both groups, *F*(3.56, 427.7) = 16.9, p < 0.001, *η*_p_^2^ = 0.12, and this decrease was steeper for the younger age group, *F*(3.56, 427.7) = 2.83, *p* = 0.030, *η*_p_^2^ = 0.02. Motivation did not differ between intention conditions (at any time point or in either age group; all *p* > 0.353). The motivational value of the monetary reward that was earned with participation in the study was higher for the younger (M 5.84, SD 1.02) than the older participants (M 3.71, SD 2.07), *F*(1,126) = 55.0, *p* < 0.001, *η*_p_^2^ = 0.30, while the older participants were more motivated by their contribution to science (M 6.37, SD 0.99) compared to the younger participants (M 5.37, SD 1.38), *F*(1,126) = 22.33, *p* < 0.001, *η*_p_^2^ = 0.15.

### 3.6. Age-related differences in cognitive measures in the lab

#### 3.6.1. Habit tendency

To measure habit tendency, participants performed a novel outcome-revaluation task, the static SORT. Performance on the SORT is described in detail in the Supplementary results S2.1 and Supplementary Figure S1. Habit tendency, operationalized as the mean of the difference scores between performance in the still-valuable versus upvalued and still-non-valuable versus devalued conditions, was significantly higher among older (M 34.3, SD 26.5) as compared to younger adults (M 4.46, SD 11.2), *t*(77.33) = −8.12, *p* < 0.001, *d* = −1.5.

However, habit tendency did not directly predict the experienced automatization of pill-taking behavior in either age group, *B* = 5.83, *p* = 0.329, or intention condition, *B* = 6.66, *p* = 0.133. Whereas the absence of a relation in younger adults could be related to their very high accuracy on the SORT and, thus, a limited range of habit tendency scores (−6.25–57.82), in older adults their more limited explicit knowledge of stimulus-outcome (S-O) associations may have affected the habit tendency measure (see Supplementary results S2.2). We therefore additionally looked into the relation between habit tendency and automatization in the selection of older adults that reported at least 6 (out of 8) truck-fruit (S-O) associations correctly after the test (*N* = 30, with 3 outliers removed). Interestingly, in this group the habit tendency score did predict the automatization that was reached at the end of the pill-taking phase, *B* = 0.55, *SE* = 0.18, *r* = 0.49, *p* = 0.006, in line with our previous findings (van de Vijver et al., 2023).

#### 3.6.2. Prospective memory

Performance on the computerized PM task is described in detail in the Supplementary results S2.2 and Supplementary Figure S2. Crucially, older adults detected fewer PM cues than younger adults did, *F*(1,120) = 21.48, *p* < 0.001, *η*_p_^2^ = 0.15, although they did show improvement across blocks: A significant interaction of age and block, *F*(1,120) = 26.36, *p* < 0.001, *η*_p_^2^ = 0.18, indicated that the number of detections increased in older adults between blocks, such that the age difference in the first PM block, *t*(120) = 6.41, *p* < 0.001, *d* = 1.16, was only at trend level in the second PM block, *t*(120) = 1.93, *p* = 0.06, *d* = 0.35. However, overall older adults had lower PM scores than younger adults on this objective task measure, in line with self-reported scores on the PRMQ.

As pill intake was higher in older than in younger adults (see section 3.2), any age-related PM decline cannot explain the current difference between age groups in pill intake. When examined per age group, the number of cue detections on the PM task also did not differ between the low pill intake group (< 27 pills) and high intake groups (> = 27 pills), younger: *t*(61) = −0.64, *p* = 0.525, *d* = −0.17, older: *t*(57) = −0.32, *p* = 0.752, *d* = −0.09. Thus, pill intake and PM task performance were also not related in the separate age groups.

#### 3.6.3. Executive functioning: working memory and task switching

As was mentioned in section 3.1, O-Span scores (reflective of WM) were lower in older than in younger adults. We did not observe a significant overall relationship between O-Span scores and pill intake group, *χ*^2^(1) = 0.054, *p* = 0.817, and there was also no significant relationship within the separate age groups (younger: *χ*^2^(1) = 0.023, *p* = 0.881; older: *χ*^2^(1) = 0.28, *p* = 0.599). We also explored the relation between O-Span scores and automatization of pill intake. Interestingly, O-Span scores were negatively associated with the level of automaticity reached at the end of the 4 weeks of pill taking, *B* = −35.7, *r* = −0.25, *p* = 0.007. When examined per age group, this effect was present in the older but not in the younger adults (younger: *B* = −10.88, *r* = −0.07, *p* = 0.576; older: *B* = −41.91, *r* = −0.29, *p* = 0.025).

Task-switching performance is described in detail in the Supplementary results S2.3 and Supplementary Figure S3. Most importantly, RTs in the switch blocks were significantly higher for switch than for stay trials in both age groups, *F*(1,123) = 93.05, *p* < 0.001, *η*_p_^2^ = 0.43 (young: *t*(66) = −10.54, *p* < 0.001, *d* = 4.70; old: *t*(57) = −6.22, *p* < 0.001, *d* = 3.66). However, the ‘switch cost’ (i.e., the difference in RTs between the two types of trials), was larger in older than in younger adults (difference: M 549.1, SD 672.0 versus M 293.2, SD 227.7), *F*(1,123) = 8.59, *p* = 0.004, *η*_p_^2^ = 0.07. Whereas switches were predictable in the regular switch blocks but unpredictable in the irregular switch blocks, the switch costs did not differ between the two types of blocks in either group (all *p* > 0.44). Interestingly, an interaction effect, *χ*^2^(1) = 9.33, *p* = 0.002, indicated that a higher switch cost was associated with lower pill intake among younger adults (low pill group: M 341.97, SD 255.27; high pill group: M 200.04, SD 119.54), *χ*^2^(1) = 8.07, *p* = 0.005, but not among older adults, *χ*^2^(1) = 1.27, *p* = 0.261.

### 3.7. Mediators of the age difference in pill intake and automaticity

We found robust age differences in both pill intake and its automaticity: Older adults took more pills than younger adults did, and also reported this behavior to be experienced as more automatic. This was not affected by the use of goal versus implementation intentions. Here, we report exploratory analyses that were performed to investigate whether age differences in pill intake and automatization were mediated by differences between older and younger adults in factors related to habit formation and motivation.

**Figure 7:**
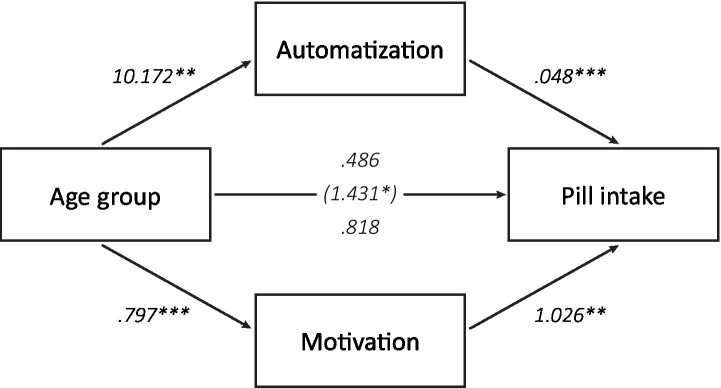
Mediating factors of age differences in pill intake. An indirect effect of age group on total pill intake was found through the average experienced automaticity during the pill-taking phase as well as through average motivation during this phase (*p < 0.05, **p < 0.01, ***p < 0.001).

To investigate whether the higher pill intake in older compared to younger adults could be explained by higher automatization, we first performed a parallel mediation analysis using PROCESS (IBM SPSS Statistics version 28.0.1.1, PROCESS version 3.5), with age group as independent variable, the number of pills that were taken as dependent variable, and average experienced automaticity during the pill-taking phase as mediating variable (*N* = 124). Because motivation to take the pills was also higher in older compared to young adults and could also explain age differences in adherence, we included average motivation during the pill-taking phase as a second mediating variable. As the age-related declines in O-Span scores, cue detections in the PM task, and switch costs in the task-switching task could not account for higher adherence in the older adults, these were not included in this mediation analysis. In line with our expectations, the indirect effect of age group on pill intake was found to be significant through experienced automatization, b = 0.486, 95% CI [0.118, 1.008] (Figure 7). A second indirect effect was found through motivation during the pill-taking phase, *b* = 0.818, 95% CI [0.262, 1.480]. Most assumptions of the mediation analysis were met, but the distributions of pill intake numbers and motivation scores were skewed because of ceiling effects. Distributions that deviate from a normal distribution do not affect the indirect effects of age group on pill intake because PROCESS uses a bootstrapping procedure to test these effects. To ensure that the separate effects in the mediation were also not influenced by the skewed distributions, we examined these effects with non-parametric, bootstrapping procedures as well. The results were almost identical to the results of the mediation analysis, indicating that the violation of the assumption of normality did not invalidate the results of the mediation analysis.

## 4. Discussion

In the current study, we investigated the role of automatic processes in the formation of a novel, daily routine in older versus younger adults. Specifically, we hypothesized that older adults are able to compensate for a decline in cognitive control functions by relying more on habit formation to form novel routines. To this end, participants were asked to take a placebo pill on a daily basis for 4 weeks, and to report their experienced automaticity of the daily intake routine every week. In line with our hypotheses, older adults adhered better to a daily (placebo) pill intake routine than younger adults, and reported higher automaticity than younger adults in the course of 4 weeks of routine formation, despite performing worse on lab measures of PM, task switching, and WM. Across age groups, automatization of pill intake was related to intake regularity and conscientiousness, but not directly to individual differences in habit tendency as measured in the lab nor to an explicit strategic planning manipulation. Importantly, the positive age effect on pill intake adherence was mediated by both motivation and automaticity. Thus, older adults were more highly motivated to adhere and this was crucial for the formation of the pill intake routine. Furthermore, they may have utilized a faster build-up of behavioral routines as compensation for their decline in cognitive control functions.

Estimates of adherence to medication regimens for chronic conditions range between 50 and 79% (DiMatteo, 2004; Briesacher et al., 2008). Adherence estimates generally do not seem to decrease, and often even show an increase, with advancing age, in both an experimental context (Park et al., 1999) and when assessed in daily life (Rolnick et al., 2013; Uchmanowicz et al., 2019). In line with these findings, in the current study older adults outperformed younger adults on pill intake adherence, and more than half of the older adults even took a (placebo) pill on all 28 days. When medication adherence does decline in older age, this is often attributed to declines in cognitive control functions such as PM (Zogg et al., 2012; Woods et al., 2014; Ihle et al., 2017), executive control, and WM (Insel et al., 2006; Smith et al., 2017). Indeed, in the current sample PM, task switching, and WM scores were lower in older compared to younger adults. However, contrary to previous findings, these declines did not result in decreased intake adherence in older adults, nor were they directly related to pill intake success. Only in younger adults, switch costs as measured with a computer task negatively predicted intake adherence. This finding may relate to the higher number of younger adults that reported being away from home as a cause of failures to adhere: with less regular circumstances, pill intake likely requires more flexible switching and adjustment.

Older adults may have compensated for their cognitive decline by resorting to other mechanisms that promote medication adherence. The current results emphasize the importance of an increased reliance on the automatization of a regularly repeating behavior as a compensatory mechanism: automatization mediated the age difference in pill intake success. Our findings align with previous studies that have demonstrated a positive relation between the self-reported habit strength of medication intake and adherence in, amongst others, oral contraceptive users (Murphy et al., 2018), HIV-positive drug users (Wagner and Ryan, 2004), and patients with asthma (Bolman et al., 2011), cystic fibrosis (Hoo et al., 2019), and hypertension (Alison Phillips et al., 2013). Furthermore, our results suggest that especially older adults may benefit from such habit formation, in line with the age-related increased reliance on habits that has been found in older adults in the lab (de Wit et al., 2014).

The automatization of a routine may depend on several factors. Importantly, for a routine behavior to become automatic and, ultimately, habitual, this behavior has to become associated with a cue or context that subsequently triggers the execution of this behavior (de Wit and Dickinson, 2009; Wood and Rünger, 2016). Context stability is therefore a key determinant of habit formation. Although we did not measure context stability directly in the current study, we reasoned that the temporal regularity of pill intake would provide us some indication. In line with our expectations, regularity indeed correlated positively with automaticity of pill intake across age groups. Furthermore, older adults showed a more regular pill intake pattern than younger adults did. This regularity may have been aided by a higher lifestyle regularity in older adults (Monk et al., 1997, 2002), which would provide more consistent contextual cues that a target behavior can be associated with.

The need of participants in both age groups to couple the action of taking the pill to a regular context is also demonstrated by the large number of participants that reported to have related pill intake to a common daily event or behavior, even when they were not instructed to do so (spontaneous planning, Bieleke and Keller, 2021). Indeed, we were not able to demonstrate beneficial effects of our strategic planning manipulation: using implementation intentions as compared to goal intentions did not increase pill intake or experienced automatization in the current study. Rather, a large part of the participants that were instructed to use a goal intention reported the use of a spontaneously conceived plan that often resembled an implementation intention, which may have been stimulated by the awareness of the participants that the current study focused on medication adherence, but also by the relative simple target behavior of taking one pill per day. Importantly, such spontaneous plans may be at least as beneficial as the implementation intentions formed prior to the study, as people might more easily remember and enact plans that are generated based on insights and their own initiative (Brickell and Chatzisarantis, 2007; Bieleke and Keller, 2021). In fact, although implementation intentions generally tend to increase successful performance of PM tasks (Gollwitzer and Sheeran, 2006), their usefulness may vary depending on the specific task and context. For example, implementation intentions are less effective in situations that place a high cognitive demand on the individual, and the use of implementation intentions does not always lead to better performance than behavioral practice (McDaniel and Scullin, 2010). Relatedly, the effects of implementation intentions are generally observed to be stronger for difficult than for easy goals (Gollwitzer and Sheeran, 2006). Future investigations therefore need to determine whether more complex medication intake schedules would benefit more from the use of implementation intentions than the relatively simple action of taking one pill a day.

Besides the influence of context stability, we also expected higher levels of subjective automatization in older compared to younger adults as a result of higher levels of habit tendency as measured in the lab, and of conscientiousness (Jackson et al., 2009; Specht et al., 2011; de Wit et al., 2014; van de Vijver et al., 2023). Although we confirmed an age-related increase in habit tendency, we did not find overall relations between habit tendency as measured in the lab and experienced automaticity of the pill intake routine. However, habit tendency did predict pill intake in the group of older adults that successfully managed to acquire the correct S-O associations in the outcome revaluation task. Older adults that did not acquire these relations would not be able to accurately adjust behavior to changing outcome values during the test phase, rendering the interpretation of their behavior in this phase more difficult. Conversely, the absence of a direct relation between experienced automaticity in the younger adults may be explained by their very high levels of behavioral success on the outcome-revaluation task, likely as a result of the changes we made to the task to make it more doable for the older adults. In contrast to habit tendency, and to previous findings, conscientiousness did not differ between younger and older adults in the current sample (Jackson et al., 2009; Specht et al., 2011). Relatedly, although conscientiousness was positively associated with the experienced automaticity of pill intake across age groups, this effect did not differ between older and younger adults. Thus, although conscientiousness seems to have benefitted routine automatization, older adults were not aided more by this than younger adults in the current study.

Our findings shed a new light on the ‘age-prospective memory paradox’ (Rendell and Craik, 2000), the differential impact of aging on performance on PM tasks in the lab and in daily life (Bailey et al., 2010; Mattli et al., 2014; Hering et al., 2016; Schnitzspahn et al., 2016). Multiple factors have been hypothesized to improve the performance of older adults outside the laboratory, including metacognitive awareness, more experience with time management, fewer distractions, better planning, and more efficient use of relevant cues (Schnitzspahn et al., 2011; Peter and Kliegel, 2018). The present study suggests that another important compensatory factor, at least for regularly repeating PM tasks, may be reliance on automatic processes for the formation of a daily routine.

Importantly, and in line with more recent explanations of the ‘age-prospective memory paradox’, our results also stress the importance of the age difference in motivation as an important determinant of real-life success in older adults (Peter and Kliegel, 2018). Indeed, younger adults have been demonstrated to reach the same performance levels as older adults on PM tasks outside the lab when being provided with extra incentives (Aberle et al., 2010). Motivation may be more important for the successful execution of PM tasks in daily life than in the lab, because it can be used to create circumstances that increase the likelihood of being able to remember the regular task at hand (e.g., coupling it to an already existing daily activity). In a computer task, circumstances are more pre-determined and cues and contexts are fixed, so even with high motivation one cannot optimize the circumstances for one’s own abilities. The fact that the personal and societal relevance of the task was emphasized before the start of the pill-taking phase, may have increased the age difference in motivation in the current study. First, because the negative impact of medication non-adherence could directly impact older adults themselves or their peers, the personal relevance of the study was likely higher in this group than in the younger adults. Additionally, the tendency towards prosocial behavior is known to show a general increase with age (Hubbard et al., 2016; Isaacowitz et al., 2021). Interestingly, the current study suggests that the increased motivation that is seen in older adults not only supports them in executing a regular behavior, but may also be related to the automatization of this behavior into a routine.

While the present study focused on the role of automatization of a daily routine, older adults may also rely on other compensation strategies to counteract the impact of their changing cognitive skills on regular PM tasks such as taking medication (Cuttler and Graf, 2007; Aronov et al., 2015; Maylor, 2018). In the current study, participants were not allowed to use any external aids to help them remember to take the pill, such as setting an alarm or writing a note, and when asked afterwards, almost none of them indicated to have done so. Still, some participants reported to have used more covert strategies that may have aided adherence, such as placing the pill bottle in a location where it would help the participant to remember taking the pill. Such compensation strategies may be especially useful in naturalistic PM tasks (Maylor, 2018; Tomaszewski Farias et al., 2018; Rummel et al., 2019), and thereby also possibly contribute to the age-prospective memory paradox, as older adults might be more accustomed to using them than younger adults (Aronov et al., 2015). Indeed, when they are given the opportunity older adults seem to rely more on intention offloading, the use of physical reminders to reduce the cognitive demand posed by the intention that has to be kept in mind, than younger adults: Older adults set more reminders for future tasks, although not to the extent that it fully compensates for their decline in prospective memory (Scarampi and Gilbert, 2021; Tsai et al., 2022). Still, the use of compensation strategies in general has been demonstrated to be positively related to higher levels of functioning in daily life in older adults (Tomaszewski Farias et al., 2018). An interesting angle for follow-up research would be to more specifically investigate the use of external aids and reminders in daily life, and age differences therein, to increase insight into who is most likely to use them and to whom this provides the largest advantage.

In line with the general trend that the use of prescription medication is higher in older age groups (Barnett et al., 2012; Mielke et al., 2020), more older than younger adults in our study reported taking medication on a daily basis. This may not only have helped them in incorporating intake of the study pill in their regular medication intake schedule or coming up with useful compensation strategies to remember to take this pill, but also suggests that they likely had more experience with regular medication intake in everyday life. Such a longer period of practice with medication intake may have eased adherence to a new medication and could also have added to the rapid automatization of this behavior. Indeed, a recent study suggests that acquiring new actions in a context in which overtraining of other, similar actions has already taken place can lead to faster conversion of the new behavior into a habit (Lesage and Verguts, 2021). Additionally, the experience with regular medication intake in older adults may have contributed to the lack of a relation between pill intake and switch costs as measured in the lab in this group. In future studies it would therefore be interesting to ask participants for how many years they have already been taking prescribed medication on a regular basis, as well as their experienced automaticity of this real-life medication intake, to examine whether have practiced the intake of one medication can speed up intake automatization of an additional one.

This study has several limitations. First of all, it is important to note that the sample of older adults in the current study originated from a WEIRD society (i.e., Western, Educated, Industrialized, Religious and Democratic; Henrich et al., 2010), was in good physical and mental health, and very conscientious and accurate in the intake of their regular medication. Therefore, this sample was not representative of the age group in general. Additionally, in the current study we only focused on taking one type of pill, at one time of day. However, as the number of health issues increases with age, so does the number of prescribed medications (Barnett et al., 2012; Charlesworth et al., 2015; Gao et al., 2018). Each of these medications has their own intake regime and some may need to be taken multiple times a day. It would be interesting to see what the role of automatization of medication intake would be in such more complex situations, where there may be multiple contexts and separate routines that need to be build. Furthermore, the question remains whether the age-related decline in cognitive control functions would be more predictive of adherence issues with more complex regimes. We should also point out that the participants in our study reached only a modest level of automaticity. While automatization of pill intake significantly contributed to intake adherence and age-related differences therein in the current study, average automatization after four weeks of pill intake was only about 60% in the older adults and even lower in the younger group. This implies that at least for a part of the participants, pill intake behavior was most likely not a (full developed) routine yet. A longer period of time, and, thus, a larger number of behavioral repetitions, may be required to fully automatize a regular behavior in daily life (Rothman et al., 2009; Lally et al., 2010). Indeed, the role of automatic processes may be even more relevant to sustain regularly repeated behaviors after longer periods of time, because this is when motivation wanes and distractions arise. Relatedly, in the current study our aim was specifically to investigate whether faster automatization in older adults may aid in pill intake adherence, and the results of our mediation analysis indeed suggest that this is the case. However, our design does not allow strong claims about the causal direction, and we acknowledge that the relation between pill intake and experienced automaticity is likely bidirectional, with repeated pill intake supporting a further increase in automaticity. While participants reached only modest levels of automatization in the current study, pill intake numbers were high and often at ceiling, which did not allow us to incorporate pill intake as a continuous measure in all analyses. We therefore chose to create a high-and low-intake group, to still be able to compare participants that showed (almost) ‘perfect’ pill intake with participants that did not, assuming that even though the low-intake group was less uniform any factors contributing to successful pill intake would likely be lower in that group than in the high-intake group. Future research will have to indicate whether the people that miss more than one pill are indeed a more vulnerable group that is more likely to continue onto a path of lower medication adherence, whether people that show a higher tendency to automatize pill intake can indeed keep their medication adherence at near-perfect levels, and how this relates to changes in cognitive functioning. Finally, in the current study participants were asked to report their intentions and experienced automaticity on a weekly basis, which may have functioned as a reminder for pill intake. Therefore, we propose that future studies should examine how routine formation and automatization of a behavior develop over longer periods of time when participants are not explicitly reminded of the behavior or the scientific purpose.

To conclude, we show that highly motivated older adults readily implement a novel routine into their daily life. Our findings stress the importance of motivation for behavior change, in line with studies demonstrating that techniques like motivational interviewing improve medication adherence (Palacio et al., 2016; Bischof et al., 2021). We suggest that such motivational techniques are an important component of behavior change interventions that aim to accomplish continued adherence over longer periods of time. Furthermore, interventions aimed at routine formation, and medication adherence in particular, may benefit from techniques that promote the automatization of the target behavior. Indeed, a meta-analysis of interventions targeted at increasing medication adherence showed the largest effect sizes for behaviorally-focused, habit-based approaches (Conn and Ruppar, 2017). Our findings suggest that increasing automatization might be achieved by stimulating a more regular intake pattern. Spontaneous strategic planning may also have supported automatization in the current study. However, further investigation of the effectiveness of strategic planning is required with proper control conditions, especially for more complex medication regimes and in less high-functioning groups. Notwithstanding the importance of continued investigation of behavior change techniques for different age groups, our current findings paint an optimistic picture of the ability to adapt one’s behavior in later life. Older adults may capitalize on relatively intact habitual processes to automatize novel routines. With motivation acting as the fuel for continued adherence and long-term habit formation, they may be even better able than younger adults to implement a novel routine.

## Data availability statement

The datasets presented in this study can be found in the online repository Figshare. Direct link: https://doi.org/10.6084/m9.figshare.23309678.

## Ethics statement

The studies involving human participants were reviewed and approved by Local Ethics Committee of the University of Amsterdam (2018-CP-9443). The patients/participants provided their written informed consent to participate in this study.

## Author contributions

IV and SW conceived the study. IV implemented the study and supervised data collection. LB and IV performed the statistical analyses. All authors contributed to the article and approved the submitted version.

## Funding

This research was supported by a VIDI grant awarded to Sanne de Wit by the Dutch Research Council (‘Nederlandse Organisatie voor Wetenschappelijk Onderzoek’ (NWO): 016.145.382).

## Conflict of interest

The authors declare that the research was conducted in the absence of any commercial or financial relationships that could be construed as a potential conflict of interest.

## Publisher’s note

All claims expressed in this article are solely those of the authors and do not necessarily represent those of their affiliated organizations, or those of the publisher, the editors and the reviewers. Any product that may be evaluated in this article, or claim that may be made by its manufacturer, is not guaranteed or endorsed by the publisher.

## Footnotes

^1^ www.qualtrics.com

^2^ https://www.lab.uva.nl/lotus/help/index.html

^3^ www.aardexgroup.com
