# Supplementary material for: Age differences in routine formation: the role of automatization, motivation, and executive functions
Source: Front Psychol. 2023 Jul 6;14:1140366. doi: 10.3389/fpsyg.2023.1140366 (PMC10357511; doi:10.3389/fpsyg.2023.1140366)
Supplement: Supplementary file 1 [file Data_Sheet_1.PDF]

**Age differences in routine formation: the role of automatization, motivation, and executive functions**

Irene van de Vijver, Lotte Brinkhof, & Sanne de Wit

## 1. Supplementary Methods

S1.1 static Symmetrical Outcome-Revaluation Task

This outcome-revaluation paradigm (based on Watson et al., 2022) consisted of a training and a test phase. On each trial of the training phase, participants were presented with a fruit truck with a colored symbol on the side accompanied by a vendor (stimulus; see Figure 2). The symbol or 'logo' indicated which type of fruit (outcome) could be obtained from that truck. Participants were supposed to only collect valuable fruits. The response window lasted 800 ms after which the vendor disappeared. Following a delay of 200 ms, the outcome fruit was presented above the truck for 700 ms. Trials were separated by a 1000-1500 ms ITI.

The task featured eight trucks, four of which appeared in the odd and four in the even blocks of the training. The four outcome fruits were presented in all blocks. Associations between trucks and outcome fruits were fixed, with each fruit being related to two trucks, one in the odd and one in the even blocks. In each block, two fruits were valuable (go condition) and two were not (no-go condition). Two fruits were always valuable in the odd, the other two in the even blocks. Thus, participants always had to press for the same trucks because they always contained the valuable fruits, which should lead to a direct S-R association between the truck and the response being formed. Before each block, the two valuable fruits were presented inside a green box and the nonvaluable fruits inside a red box for four sec. Next, the participant saw all fruits and had to indicate using the mouse which two had to be collected. If the response was incorrect, the presentation and check were repeated. Importantly, during the blocks participants did not receive performance feedback but had to assess the accuracy of their (non-)responses themselves using their knowledge of the current values of the fruits.

During the test phase, the same procedure preceded each block and participants were still supposed to only collect the valuable fruits by pressing for the associated trucks. However, during the trials the outcome fruits were now replaced with a general picture of a fruit crate, so participants had to rely on their previously learned associations between trucks and fruits. Moreover, each block now featured all eight trucks. Per block, a different combination of four outcome fruits was valuable, which was never the same as during training. Because specific truck-fruit combinations were always (non)valuable during training, the combination of the training value and the new values in the test

resulted in two truck-fruit combinations being still valuable, two being devalued, two being upvalued, and two being still nonvaluable in each block.

During training, in each block all four trucks were presented four times, twice in the first and twice in the second half. Trial order was randomized per eight trials. The distribution of trucks and fruits over blocks and conditions was randomized over participants. The training phase consisted of 32 blocks. After every 8 blocks, participants could take a self-paced break of maximally 30 sec. The test phase consisted of four blocks. All trucks were again presented four times per block, twice in each half, and trials were now randomized per 16. In both phases, collecting a valuable fruit resulted in the gain of 1 point, collecting a nonvaluable fruit in the loss of 1 point. After each block, the numbers of correct button presses, correct non-responses, and late responses, as well as the total number of points collected so far were presented on screen for 5 sec. Before starting the real task, participants receive extensive instructions and practiced the training (two blocks) and test phase (one block) with task-irrelevant trucks and fruits. They were also informed that the participant per age group that gained the most points would receive two cinema vouchers.

Between the training and the test, the explicit knowledge of the truck-fruit associations was assessed. Each truck was shown in combination with all fruits. The participant has to select which fruit was sold by that truck, and indicate his/her confidence in the judgement on a color bar ranging from red ('very uncertain') to green ('very certain').

After finishing the test phase, participants performed a second, 'baseline' test. This test phase was mostly identical to the first test phase, but now in each block four stimuli rather than outcomes were devalued. Participants were now instructed not to respond to the devalued stimuli. This part was incorporated to check whether age differences in responding in the test phase could not be attributed to general difficulties with implementing a devaluation and changing the response pattern accordingly. Finally, participants filled out a questionnaire about strategy use during the task, and whether they focused more on the fruits or trucks and on pressing or not pressing. Completion of all parts of the SORT took approximately 75 minutes in total.

For all behavioral analyses, we focused on two measures: response accuracy during training in the go and no-go conditions, and response accuracy during the test and baseline test in the still valuable, devalued, upvalued, and still nonvaluable conditions.

S1.2 Prospective Memory task

Participants performed a lexical decision task (LDT) with additional PM assignments (similar to Ihle et al., 2018). On each trial of the LDT, they had 1500 ms to indicate whether a 5-letter string was a word or non-word by pressing a left or right key ('E' and 'O', mapping to words and non-words

counterbalanced over participants; see Figure 3a). Half of the trials featured words, the other half non-words. Trials were separated by a 1000 ms inter-trial interval (ITI).

After a practice block (30 trials) and a first test block consisting only of the LDT (108 trials), in a second and third test block a PM assignment was added. Specifically, in each block two cue words were presented six times intermixed with the 108 LDT trials, resulting in 120 trials per block. Presentations of cue words in a block were quasi-randomly distributed: they had to be separated by at least 2 non-cue strings and could not feature in the first five trials. If participants saw a cue word they had to press the space bar. The cue words were associated with specific action words that the participants were instructed on before the block started. For example, two instructions could be 'The CANDY is in the KITCHEN' and 'The CHIPS are in the BASEMENT' (instructions in the other block were about getting an umbrella or boots when there was rain or hail). After pressing the space bar for the candy or chips cue and a delay of 300 ms, the possible action words were displayed on both sides of the screen. The participant now had to indicate the action related to the cue by pressing the shift key on the corresponding side (no time limit). After a warning that the regular task was about to continue (2000 ms), the next string appeared. The PM task including instructions and practice took 20 minutes for the younger adults and 30 minutes for the older adults to complete.

Eight participants (four young) had no (complete) data for the PM task and one young adult had a very low accuracy score and was considered an outlier. Their data were therefore not included in the analyses of PM behavior. For the analyses of task behavior, we examined response accuracy and reaction times (RT) on the ongoing, lexical decision task, as well as the number of correctly detected PM cues and the RTs of these detections. Participants that did not correctly detect at least one PM cue in each block were excluded from the RTs analyses.

### S1.3 Task-switching task

On each trial participants saw a cue consisting of a letter and a number (adapted from Sohn, Ursu, Anderson, Stenger, & Carter, 2000). Presentation sides of the letters and numbers were counterbalanced over trials. Depending on the color of the cue, the participant had to indicate either whether the digit was odd (1, 3, 5, 7) or even (2, 4, 6, 8), or whether the letter was a vowel (A, E, O, U) or a consonant (R, B, N, H; the meaning of the colors was counterbalanced over participants). There was no time limit to the response window. 1000 ms after the response a new digit-letter combination was presented.

The task contained four types of blocks. In the number and letter blocks the color of the cues was constant and participants had to focus only on the numbers or only on the letters. In the regular switch blocks, the cue color switched every two trials, so participants could predict when to switch

from focussing on the number to the letter and vice versa. In the irregular switch blocks, the order of the colors was unpredictable and the participant could only rely on this color to know the assignment. Participants were notified before each block of the upcoming block type. Blocks consisted of 25 trials. Each block type was presented four times, resulting in a total of 16 blocks. Block order was randomized per four blocks, while avoiding direct repetitions. Before the real task, participants practiced all four block types. Completion of the task-switching task took about 30 minutes for the younger and 35 minutes for the older adults.

For all analyses, the first trial of a block was disregarded, since this could never be a repetition or switch. The overall switch cost, defined as the average of the difference in RT between stay trials (same rule had to be applied) and switch trials (switch from number to letter rule or vice versa) in the regular and irregular switch blocks, was used for further analyses.

S1.4 Adapted Social Rhythm Metric short form

Lifestyle regularity was assessed with an adaptation of the short Social Rhythm Metric (SRM-5; Monk et al., 2002). The SRM-5 is a diary instrument to measure regularity in the timing of five daily events. Participants are supposed to record every evening when and with whom these events took place on that day. To avoid that the daily recording would turn into a reminder for pill intake, we refrained from asking participants to fill out the SRM-5 every day. Instead, we asked them in the lab to estimate for each of the five items (1) the time on which this event usually took place in the previous month, and (2) on how many days in that month the event had taken place in a 1,5 hour interval surrounding this time. Unfortunately, both unexpected response patterns and verbal report from participants indicated that some participants erroneously interpreted the second question as how often the event had *not* taken place in the 1,5 hour interval. Therefore, the data from this adapted questionnaire had to be considered unreliable and were excluded from further analyses.

## 2. Supplementary Results

S2.1 Habit propensity in the lab

During the training phase of the static symmetrical outcome revaluation task (SORT), both younger and older adults learned to press for trucks containing valuable outcome fruits and not press for trucks containing non-valuable outcome fruits. A mixed-design ANOVA with factors age group, block, and value indicated that accuracy increased over blocks, $F(4.85, 610.6) = 204.3$, $p < .001$, $\eta_p^2 = .619$, although this increase was faster in younger compared to older adults, $F(4.85, 610.6) = 34.21$, $p < .001$, $\eta_p^2 = .214$ (see Figure S1a). Interestingly, an interaction effect of age group and value, $F(1,126) = 47.48$, $p < .001$, $\eta_p^2 = .274$, indicated that whereas younger adults performed equally well for valuable and non-valuable outcomes (i.e., on Go and NoGo trials), $t(67) = -0.35$, $p = .731$, $d = -0.05$, older adults showed a higher accuracy for non-valuable compared to valuable outcomes, $t(59) = -6.67$, $p < .001$, $d = -1.21$, although this difference decreased over blocks, $F(3.81, 480.7) = 27.66$, $p < .001$, $\eta_p^2 = .180$.

The analysis of the critical test phase, using a mixed-design ANOVA with factors age group, test value, and congruency with training, revealed a significant congruency effect, suggesting the presence of a revaluation effect, $F(1,126) = 121.3$, $p < .001$, $\eta_p^2 = .49$ (Figure S1b). Whereas a significant interaction with age group indicated that this effect was larger in older compared to younger adults, $F(1,126) = 71.9$, $p < .001$, $\eta_p^2 = .36$, the main effect was significant in both groups (younger: $F(1,67) = 10.8$, $p = .002$, $\eta_p^2 = .14$, older: $F(1,59) = 100.7$, $p < .001$, $\eta_p^2 = .63$). Indeed, in both age groups, accuracy was higher for still valuable than upvalued (younger: $t(67) = 2.62$, $p = .011$, $d = 7.95$, older: $t(59) = 9.44$, $p < .001$, $d = 2.38$), and for still non-valuable than devalued outcomes (younger: $t(67) = 3.28$, $p = .002$, $d = 16.3$, older: $t(59) = 7.06$, $p < .001$, $d = 3.54$). An interaction of age group, test value, and congruency, $F(1,126) = 14.6$, $p < .001$, $\eta_p^2 = .10$, indicated that the revaluation effect was most pronounced in older adults when a response was required. Indeed, younger adults performed equally well for still-valuable and still-non-valuable outcomes, $t(67) = 0.42$, $p = .678$, $d = 17.7$, in line with their comparable accuracy for go and no-go conditions during the training. They also showed a similar performance for upvalued and devalued outcomes, $t(67) = 0.84$, $p = .403$, $d = 7.62$, suggesting they were equally effective in both types of behavioral adjustment. In older adults, performance was higher for still-non-valuable compared to still-valuable outcomes, $t(59) = 7.75$, $p < .001$, $d = 3.93$, which also mirrors their higher performance in the no-go compared to the go condition during training. However, their accuracy was much lower for upvalued compared to devalued outcomes, $t(59) = -10.3$, $p < .001$, $d = 2.31$.

In the subsequent baseline test, in which stimuli rather than outcomes were devalued, younger adult also were more accurate than older adults, $F(1,126) = 51.6$, $p < .001$, $\eta_p^2 = .29$ (younger: M 96.7, SD 5.35, older: M 89.8, SD 10.5; Figure S1c). An interaction effect of age group and test,

$F(1,126) = 21.5$, $p < .001$, $\eta_p^2 = .15$, indicated that whereas performance of younger adults did not differ between valuable and non-valuable stimuli during this test, $t(67) = -1.76$, $p = .082$, $d = -0.20$, performance of older adults was again higher for non-valuable than valuable stimuli, $t(59) = -5.66$, $p < .001$, $d = -0.83$. Whereas performance on this baseline test does not depend on outcome knowledge, it can still depend on previously learned S-R relations. In line with this idea, a main effect for congruency, $F(1,126) = 5.9$, $p = .016$, $\eta_p^2 = .04$, showed that accuracy was higher for stimuli associated with outcome values that were congruent as compared to incongruent with the values during training. There were no other significant effects of the previously learned associations (all p-values > .12).

The higher accuracy for non-valuable outcomes that older adults showed during training and test seems to suggest that they focused on learning the S-O associations and related responses in the no-go rather than the go conditions. However, an alternative explanation could be that the general response tendency in older adults was to refrain from responding. Indeed, whereas both optimal and completely random behavior would result in a response rate of ~50%, the overall response rates in older adults were only 44.2% (SD 6.73) during training and 33.7% (SD 10.6) during test. This was significantly lower than the response rates in the younger adults during both training (49.9%, SD 1.30; $t(126) = 6.89$, $p < .001$, $d = 1.22$) and test (49.6%, SD 6.49; $t(126) = 10.4$, $p < .001$, $d = 1.84$). Such a low response rate would lead to seemingly optimal behavior in the no-go conditions without acquiring any knowledge about the S-O relations or related responses. This would also explain the higher accuracy for non-valuable stimuli that older adults demonstrated during the baseline test.

This explanation was confirmed by the explicit assessments of S-O knowledge that were obtained after the test phase. A mixed-design ANOVA with factors age group and value showed that S-O knowledge was generally higher in younger compared to older adults, $F(1,126) = 60.33$, $p < .001$, $\eta_p^2 = 0.32$, and that the effect of value differed between the groups, $F(1,126) = 18.36$, $p < .001$, $\eta_p^2 = 0.13$. The difference in knowledge of S-O associations for outcomes that were valuable versus non-valuable during training was only at trend level for younger adults, $t(67) = 1.73$, $p = .09$, $d = 4.62$, and knowledge was high in both conditions. In older adults, however, knowledge of associations for outcomes that were valuable during training was much higher than for outcomes that were non-valuable, $t(59) = 5.01$, $p < .001$, $d = 1.44$ . Thus, the problems that older adults experienced during the test phase were not only due to a difficulty in response adjustment, but also a lack of knowledge of the correct S-O associations, and, thus, the appropriate behavior, especially in the upvalued condition.

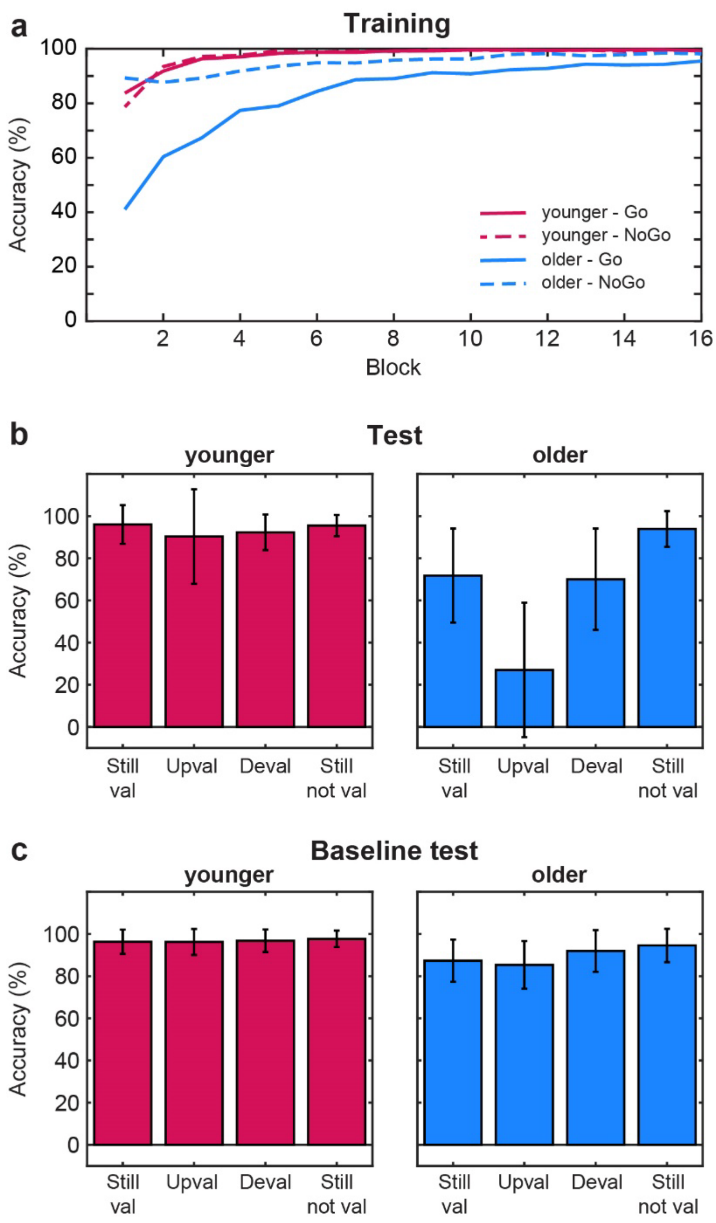


**Figure S1. Age differences in habit propensity.** (a) Accuracy in the training phase increased faster in younger than older adults. Older adults showed a higher accuracy for non-valuable compared to valuable outcomes. (b) In the test phase, the difference in accuracy between responding to stimuli with training-congruent and training-incongruent outcome values, or the 'revaluation effect', was present in both age groups, although it was larger in older compared to younger adults. In older adults the revaluation effect was most pronounced when a response was required. (c) During the baseline test, accuracy was higher in younger compared to older adults, and for stimuli associated with training-congruent as compared to training-incongruent outcome values. Older adults again showed higher accuracy for non-valuable compared to valuable outcome values (error bars represent standard deviations; Still val = still valuable, Upval = upvalued, Deval = devalued, Still not val = Still not valuable).

S2.2 Lab measures of prospective memory

Behavior on the computerized PM task was analyzed with mixed-design ANOVAs with factors age group and block. Overall LDT accuracy (accuracy on the ongoing task) did not show an effect of age, $F(1,120) = 1.25$, $p = .266$, $\eta_p^2 = 0.01$, but older adults were significantly slower than younger adults, $F(1,120) = 83.27$, $p < .001$, $\eta_p^2 = 0.41$ (see Figure S2a and S2b). For both accuracy and RT on the LDT, we found a significant interaction between age group and block (accuracy: $F(1.82, 217.9) = 17.17$, $p < .001$, $\eta_p^2 = 0.13$, RT: $F(1.93, 231.1) = 8.93$, $p < .001$, $\eta_p^2 = 0.07$. Pair-wise comparisons revealed that younger adults performed better, both p-values < .006, but slower, both p-values < .001, in both blocks in which PM cues were added than in the single-task block, likely due to practice effects. In older adults, performance in the first PM block was worse as well as slower than in the single-task block, both p-values < .001, but in the second PM block accuracy returned to the original level, $p = .165$, although RTs remained slower, $p < .001$.

A similar pattern was seen in the number of PM cue detections. The average number of cue detections across both blocks was higher for younger than older adults, $F(1,120) = 21.48$, $p < .001$, $\eta_p^2 = 0.15$ (see Figure S2c). However, a significant interaction of age and block, $F(1,120) = 26.36$, $p < .001$, $\eta_p^2 = 0.18$, indicated that the number of detections was higher for younger compared to older adults in the first PM block, $t(120) = 6.41$, $p < .001$, $d = 1.16$, but the number of detections increased in older adults between blocks such that there was no age difference anymore in the second PM block, $t(120) = 1.93$, $p = .06$, $d = 0.35$. Younger adults were also faster to respond to PM cues than older adults, $F(1,114) = 86.43$, $p < .001$, $\eta_p^2 = 0.43$ (see Figure S2d; N=116 after excluding participants without sufficient detections). This effect did not differ between blocks, $F(1,114) = 1.21$, $p = .273$, $\eta_p^2 = 0.01$. Additionally, after responding to the PM cues younger adults (M 97.4%, SD 10.7) were better able than older adults (M 88.0%, SD 24.0) to select the correct action in relation to the PM cue in both blocks, $F(1,114) = 11.50$, $p < .001$, $\eta_p^2 = 0.09$ (other p-values > .098).

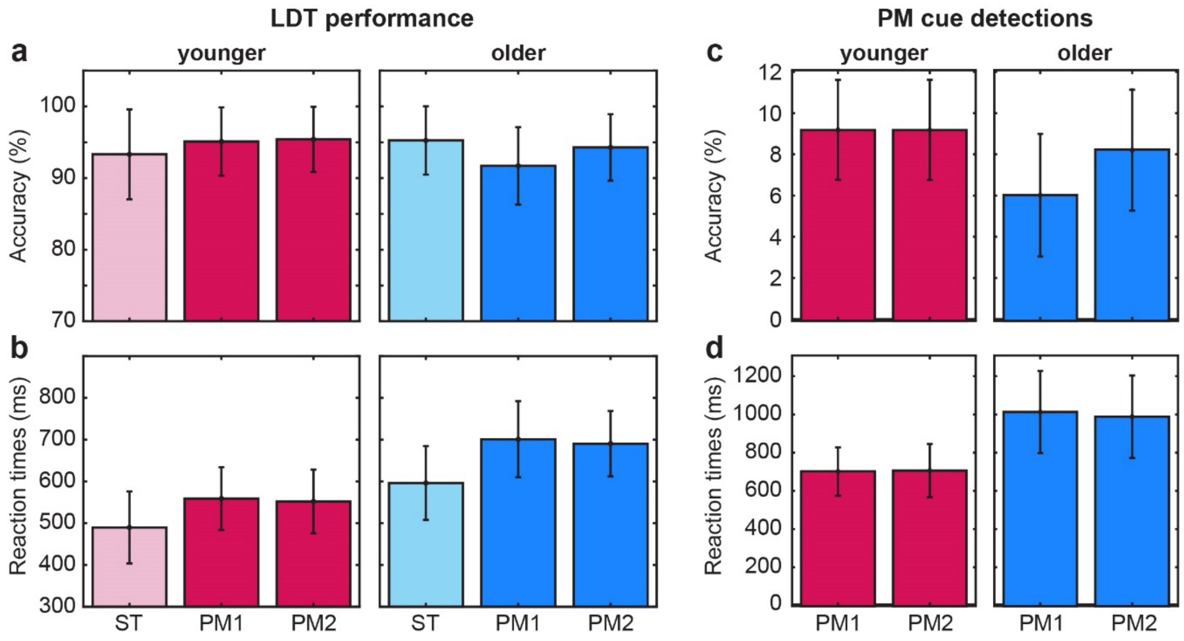


**Figure S2. Prospective memory performance in the lab.** (a) Younger adults performed better on the Lexical decision task (LDT) in both blocks in which prospective memory (PM) cues were added than in the single-task block (ST). In older adults, performance in the first PM block was worse than in the single-task block but in the second PM block accuracy returned to the original level. (b) Both age groups responded slower in the blocks in which PM cues were added compared to the single-task block. (c) The number of PM cue detections was higher for younger compared to older adults in the first but not in the second PM block. (d) Younger adults responded faster to PM cues than older adults (error bars represent standard deviations).

S2.3 Task switching

Younger adults performed better, $F(1,123) = 4.67$, $p = .03$, $\eta_p^2 = 0.04$, as well as faster, $F(1,123) = 75.5$, $p < .001$, $\eta_p^2 = 0.38$, than the older adults on the single-task blocks of the task-switching task (see Figure S3a and S3b). Accuracy and RT in the switching blocks were analyzed with mixed-design ANOVAs with factors age group, block (stay, switch), and trial type (regular, irregular). In these blocks, overall accuracy was also higher for younger (M 0.95, SD 0.06) than older adults (M 0.89, SD 0.15), $F(1,123) = 11.83$, $p < .001$, $\eta_p^2 = 0.09$, as well as for regular (M 0.93, SD 0.12) compared to irregular blocks (M 0.92, SD 0.11), $F(1,123) = 5.58$, $p = .02$, $\eta_p^2 = 0.04$, and for stay (M 0.93, SD 0.11) compared to switch trials (M 0.92, SD 0.12), $F(1,123) = 12.13$, $p < .001$, $\eta_p^2 = 0.09$ (Figure S3c). The effect of block type differed between stay and switch trials, $F(1,123) = 3.93$, $p = .05$, $\eta_p^2 = 0.03$: While accuracy on stay trials was higher in the regular than irregular blocks, $t(124) = 3.22$, $p = .002$, $d = 1.16$, no such differences were found for the switch trials, $t(124) = 0.56$, $p = .575$, $d = 1.14$. There were no other significant interactions between age, block type or trial type (all p-values > .31).

RTs in the switching blocks were, in line with the accuracy results, higher for older (M 2025.9, SD 907.6) compared to younger adults (M 1055.0, SD 406.8), $F(1,123) = 85.34$, $p < .001$, $\eta_p^2 = 0.41$, for irregular (M 1559.8, SD 788.4) compared to regular blocks (M 1451.2, SD 886.0), $F(1,123) = 22.95$, $p < .001$, $\eta_p^2 = 0.16$, and for switch (M 1711.5, SD 969.0) compared to stay trials (M 1299.5, SD 623.1), $F(1,123) = 93.05$, $p < .001$, $\eta_p^2 = 0.43$ (Figure S3d). However, the difference between regular and irregular blocks differed between age groups, $F(1,123) = 35.73$, $p < .001$, $\eta_p^2 = 0.23$, with no difference among older adults, $t(57) = 0.755$, $p = .459$, $d = 3.58$, but lower RTs among younger adults in the regular compared to the irregular blocks, $t(66) = -8.60$, $p < .001$, $d = 4.75$. The difference between stay and switch trials also differed between age groups, $F(1,123) = 8.59$, $p = .004$, $\eta_p^2 = 0.07$: in both age groups RTs were significantly longer for switch than for stay trials, young: $t(66) = -10.54$, $p < .001$, $d = 4.70$; old: $t(57) = -6.22$, $p < .001$, $d = 3.66$, but this difference was larger in older than in younger adults (difference old: M 549.1, SD 672.0, difference young: M 293.2, SD 227.7).

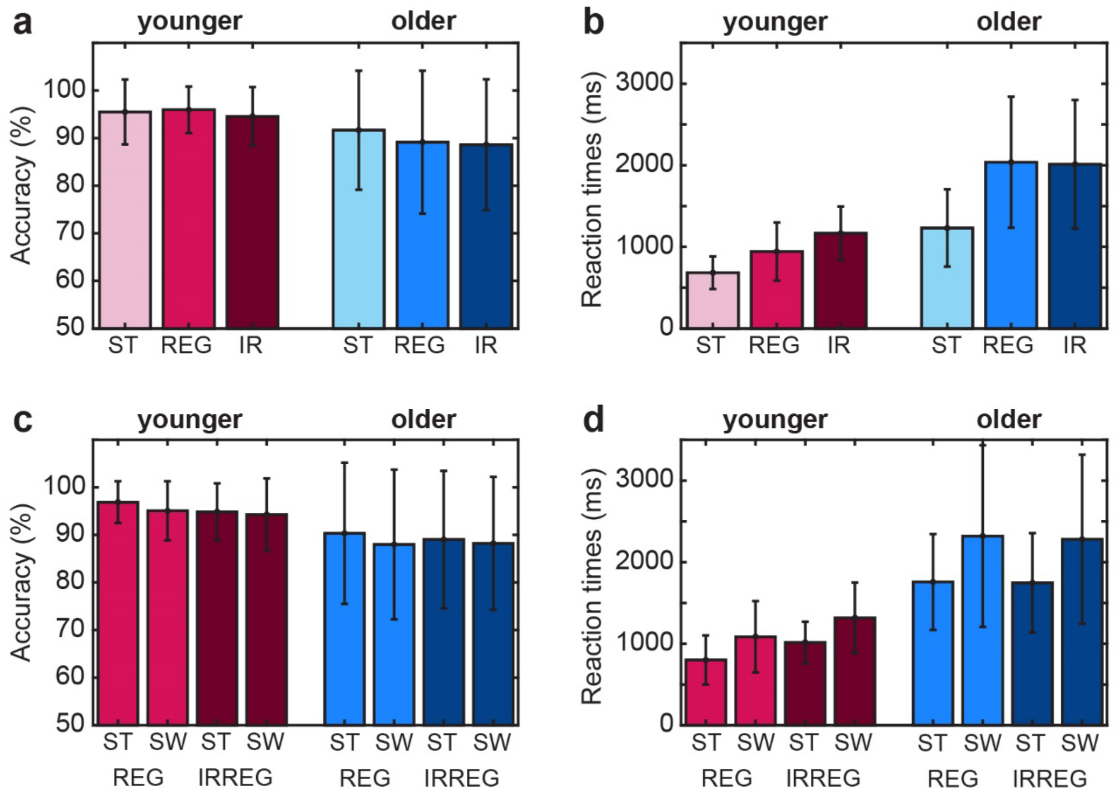


**Figure S3. Task-switching behavior in younger and older adults.** (a) Task accuracy was higher and (b) reaction times were lower in younger compared to older adults. (c) In the switching blocks, accuracy was higher in regular (REG) compared to irregular (IRREG) blocks and on stay (ST) compared to switch (SW) trials. Accuracy on stay but not on switch trials was higher in the regular than the irregular blocks. There were no interactions with age group. (d) Only for younger adults, RTs were faster for regular compared to irregular blocks. RTs were faster for stay compared to switch trials in both age group, but the difference was larger in older adults (error bars represent standard deviations; ST = single-task block).

**References**


Singmann, H., Bolker, B., Westfall, J., Aust, F., & Ben-Shachar, M.S. (2021). afex: Analysis of Factorial Experiments. In *baselr.org* (p. 77).

Ihle, A., Albiński, R., Gurynowicz, K., & Kliegel, M. (2018). Four-Week Strategy-Based Training to Enhance Prospective Memory in Older Adults: Targeting Intention Retention Is More Beneficial than Targeting Intention Formation. *Gerontology*, *64*(3), 257–265. https://doi.org/10.1159/000485796

Monk, T. H., Frank, E., Potts, J. M., & Kupfer, D. J. (2002). A simple way to measure daily lifestyle regularity. *Journal of Sleep Research*, *11*(3), 183–190. https://doi.org/10.1046/j.1365-2869.2002.00300.x

Sohn, M. H., Ursu, S., Anderson, J. R., Stenger, V. A., & Carter, C. S. (2000). The role of prefrontal cortex and posterior parietal cortex in task switching. *Proceedings of the National Academy of Sciences of the United States of America*, *97*(24), 13448–13453. https://doi.org/10.1073/pnas.240460497

Watson, P., Gladwin, T. E., Verhoeven, A. A. C., & de Wit, S. (2022). Investigating habits in humans with a symmetrical outcome revaluation task. *Behavioral Research Methods*. https://doi.org/10.3758/S13428-022-01922-4
